# Systemic proteome remodeling in soybean during early nodulation: distinct responses in roots and leaves

**DOI:** 10.3389/fpls.2026.1952260

**Published:** 2026-09-16

**Authors:** Afrodite Katsaouni, Emma Guillierme, Sander E. Van der Verren, Kris Gevaert, Sofie Goormachtig, Sylwia Struk

**Affiliations:** 1 Department of Plant Biotechnology and Bioinformatics, Ghent University, Ghent, Belgium; 2 VIB Center for Plant Systems Biology, VIB, Ghent, Belgium; 3 Department of Biomolecular Medicine, Ghent University, Ghent, Belgium; 4 VIB Center for Medical Biotechnology, VIB, Ghent, Belgium

**Keywords:** autoregulation of nodulation (AON), early nodulation, immunity, isoflavonoids, metabolomics, NARK receptor, proteomics, soybean

## Abstract

Here, we aimed to deepen our understanding of the integration between root and shoot signaling during early nodulation by jointly analyzing proteome-level dynamics in roots and leaves of the wild type (WT) and the hypernodulating autoregulation-of-nodulation (AON) mutant, *nark*. To this end, we performed a time-course shotgun proteomics experiment in soybean roots and leaves of the same plants inoculated with *Bradyrhizobium diazoefficiens.* In WT roots, we observed proteome changes linked to calcium-dependent signaling, cellular reorganization, and nutrient allocation. In the leaves, isoflavonoid biosynthesis and immunity-associated responses were differentially regulated in the WT and *nark* mutant during nodulation. We hypothesize that AON signaling involves a complex interplay between isoflavonoids and immunity, which might be associated with jasmonic acid and/or systemic auxin transport.

## 1 Introduction

Soybean (*Glycine max*) stands out as one of the most economically significant legume crops, serving as an essential and cost-effective source of protein and oil (Ferguson et al., 2019; Karges et al., 2022). The nitrogen required to sustain the intrinsic richness of soybean seeds in proteins and lipids can be obtained through a mutualistic symbiosis with diazotrophic rhizobia, which takes place in *de novo* formed root organs known as nodules. Within these nodules, rhizobia convert atmospheric nitrogen (N_2_) into ammonia, a form of nitrogen readily assimilated by the plant (Ferguson et al., 2010, 2019; Zhong et al., 2024). This legume–rhizobia symbiosis is tightly regulated through a complex molecular dialogue between host and bacteria, and elucidating its control could improve nitrogen-use efficiency and crop production and/or quality (Oldroyd, 2013; Ferguson et al., 2019).

Nodulation is initiated under low nitrogen conditions, prompting legumes to secrete specific flavonoid blends that attract compatible rhizobia and induce the production of specific lipo-chitooligosaccharide signals, known as Nod factors (NFs) (Dénarié et al., 1996; Ferguson et al., 2010). NFs are recognized by specific receptor kinases in the root epidermis, the Nod factor receptor 1 (NFR1) and NFR5, initiating signaling events to which nuclear calcium oscillations are central. These are subsequently decoded by the calcium- and calmodulin-dependent protein kinase DOESN’T MAKE INFECTIONS3 (DMI3, also known as CCaMK), allowing downstream responses that lead to bacterial infection and nodule organogenesis (Ferguson et al., 2010; Yang et al., 2022a). In many legumes, upon NF recognition, root hairs curl to entrap rhizobia, which are guided into *de novo* made subepidermal nodule primordia via infection threads, where they differentiate into nitrogen-fixing bacteroids (Ferguson et al., 2010; Yang et al., 2022a). Besides this pathway, alternative nodulation mechanisms have been described including intercellular infection (e.g. crack entry) and NF-independent symbioses across different legumes and nodulating species (Okazaki et al., 2013; Araújo et al., 2025).

To balance symbiosis and plant growth, legumes control the optimal number of nodules through a systemic autoregulation of nodulation (AON) pathway, entailing a negative feedback mechanism induced by rhizobia infection (Ferguson et al., 2019; Okuma and Kawaguchi, 2021). In soybean, the AON pathway is initiated by the production of the rhizobia-induced CLAVATA3/Embryo Surrounding Region-Related (CLE) peptides RIC1 and RIC2 in the roots, which are then post-translationally tri-arabinosylated and transported through the xylem to the shoot (Lim et al., 2011). There, they are perceived by the nodulation autoregulation receptor kinase (NARK), leading to its activation and possible phosphorylation of downstream targets. NARK is expressed in phloem tissues and was shown to interact with and phosphorylate the kinase-associated protein phosphatases (KAPP) 1 and KAPP2, whereafter they dephosphorylate NARK to attenuate the signal (Nontachaiyapoom et al., 2007; Lim et al., 2011; Chaulagain and Frugoli, 2021; Pereira et al., 2024). The hypernodulation phenotype of *nark* mutants is indicative of the central role of NARK in the regulation of nodulation. The signaling events downstream of NARK are not fully elucidated, yet one identified secondary signal is microRNA2111 (miR2111) (Zhang et al., 2021). In soybean, miR2111 was demonstrated to translocate to the root and trigger the degradation of mRNAs encoding the Kelch repeat-containing F-box proteins Too Much Love (TML) 1a, TML1b, and TML2, which are predicted to be involved in ubiquitination and subsequent 26S proteasomal degradation of targets (Magori et al., 2009; Takahara et al., 2013; Grumati and Dikic, 2018). When the AON pathway is active, the miR2111 transcript levels decrease, leading to TML protein accumulation in the roots and nodulation inhibition via currently unknown mechanisms (Okuma and Kawaguchi, 2021). In addition to the miR2111–TML module, other systemic signals have been proposed, including transcriptional repression of *NFR* genes and interactions with cytokinin and jasmonic acid (JA) signaling pathways, although these effects appear to be context-dependent and are less well defined mechanistically (Seo et al., 2007; Kinkema and Gresshoff, 2008; Gamas et al., 2017; Tsikou et al., 2018; Gautrat et al., 2019).

To better understand these nodulation processes at the protein level, proteomic approaches provide a complementary perspective. Proteomic studies are less commonly applied than transcriptomic approaches due to their comparatively lower sensitivity, but they offer valuable insights into the wide array of proteins that ultimately shape cellular physiology. For nodulation, previous proteomic studies mainly focused on targeted proteomic analyses of specific tissues, such as root hairs, nodules, or symbiosomes, to uncover protein changes during nodule development, resulting in the identification of various metabolic, defense, and signaling proteins associated with infection and nitrogen fixation (Lim et al., 2010; Nguyen et al., 2012; Salavati et al., 2012; Clarke et al., 2015; Lyu et al., 2022b). Proteomic approaches to study systemic signaling like AON are limited and have focused on specific compartments such as xylem sap, identifying candidate mobile proteins involved in long-distance signaling (Subramanian et al., 2009). However, these approaches provide only a partial view of the dynamic root–shoot communication underlying AON. To address this, we employed an untargeted proteomics strategy using both root and shoot tissues to uncover novel components regulating nodulation initiation and control. Whereas root proteomics were used to characterize proteome changes associated with early nodulation, leaf proteomics specifically focused on systemic signaling responses involved in autoregulation of nodulation (AON). To distinguish AON-associated responses from general systemic responses, the AON-defective mutant *nark* was included in the leaf proteomic analysis.

## 2 Materials and methods

### 2.1 Growth conditions for plants and bacteria

Soybean cultivar Williams82 (Wm82) and the *nark* mutant *nod4* (Pracht et al., 1993), kindly provided by prof. Brett Fergusson (University of Queensland, Australia), were used in this study. Seeds were gas sterilized, pregerminated in the dark (24 °C, 4 days) and subsequently transferred to an aeroponics system filled with the nitrogen-poor nutrient solution Soli (**Supplementary Table S1**) (24 °C, 16 h:8 h light:dark). After two weeks of growth, the plants were inoculated with the labelled *Bradyrhizobium diazoefficiens* (*B. diazoefficiens*) bjGFP strain, kindly provided by prof. Hans-Martin Fischer (ETH Zurich, Switzerland) (Ledermann et al., 2015). Specifically, *B. diazoefficiens* bjGFP was grown in YMA (1 g/l yeast extract, 10 g/l D-mannitol, 0.50 g/l K_2_HPO_4_, 0.20 g/l MgSO_4_·7H_2_O, 0.10 g/l, NaCl, 20 g/l agar) at 28 °C under rotation until an OD600 of 0.8–1 was reached. The bacteria were resuspended in Soli and diluted 1000x in the aeroponics system.

### 2.2 RNA extraction and RT-qPCR

Plant roots and leaves were harvested every morning between 0 and 7 days post-inoculation (dpi) and at 10 dpi and 14 dpi, this was repeated four times independently to generate four biological replicate experiments. Tissues from 3 to 5 plants were pooled per timepoint. For leaf tissue, all trifoliate leaves were collected, while for root tissue, the whole root was collected excluding the root tip. Samples were ground into a fine powder, and RNA was extracted with the ReliaPrep™ kit (Promega) according to the manufacturer’s instructions. RNA quantity and quality were measured with NanoDrop One (Thermo Scientific™). Then, one µg of RNA was converted into cDNA using the qScript cDNA synthesis mix (QuantaBio). For RT-qPCR analysis, all measurements were done in three technical replicates in 384‐multiwell plates and averaged for further calculations. Reaction mixes were composed by the Janus Robot (PerkinElmer) with a final volume of 5 μL and a 10% cDNA fraction with the SYBR^®^Green Master Mix (PerkinElmer). The RT‐qPCR experiments were performed by a Lightcycler^®^ 480 II (Roche Diagnostics) with the following settings: 1x preincubation (95 °C for 5 min), 45x amplification (95 °C for 10 s, 60 °C for 10 s, and 72 °C for 10 s), 1x melting curve (95 °C for 5 s, 65 °C to 97 °C for 1 min), and 1x cooling down (40 °C for 10 s). Cycle threshold values were determined by the Lightcycler^®^ 480 software and analyzed using the 2^−ΔΔCt^ method (Livak and Schmittgen, 2001). The relative expression data were normalized to the expression levels of *GmATP synthase* (Glyma.20G123600) (Reid et al., 2011a) and *GmCons6* (Glyma.12G051100) (Libault et al., 2008). Sequences of all primers used in this study are provided in **Supplementary Table S2**. Statistical significance was evaluated with Welch Anova and Games-Howell *post-hoc* analysis (Games and Howell, 1976).

### 2.3 Protein extraction and mass spectrometry

Plant roots (excluding the root tip) and leaves were harvested at 0, 3 and 7 dpi. For each genotype and timepoint, tissues from 5–7 plants were pooled to obtain a representative sample and to reduce biological variability arising from asynchronous nodulation events among individual plants, as observed during preliminary experiments and RT-qPCR analyses. In addition, systemic nodulation responses are thought to localize primarily in vascular tissues, which represent only a small fraction of the total root and leaf tissue analyzed here (Okamoto et al., 2013; Zhang et al., 2021; Song et al., 2022). Consequently, biologically relevant responses may be diluted when whole organs are analyzed. After pooling, each sample was ground into a fine powder and divided into three technical replicates that were processed independently for protein extraction, sample preparation and LC-MS/MS analysis. These technical replicates originated from the same pooled biological sample and were used to assess technical variation; no independent biological replicates were included in the proteomics experiment.

Protein extraction was performed according to the previously described procedure from Zhu et al. (2024) with minor modifications. In brief, 0.3 g of finely ground plant material was suspended in homogenization buffer containing 30% sucrose, 50 mM Tris-HCl buffer (pH 8), 0.1 M KCl, 5 mM EDTA, and 500 mM DTT in Milli-Q water, to which the appropriate amounts of the cOmplete™ protease inhibitor mixture (Roche) and the Pierce™ Phosphatase Inhibitor mini tablets (Thermo Scientific™) were added. The samples were sonicated for 30 s on ice and centrifuged at 4 °C for 15 min at 3,220 g to remove debris. Supernatants were collected and a methanol/chloroform precipitation was carried out by adding 3, 1, and 4 volumes of methanol, chloroform and water, respectively. Samples were centrifuged for 15 min at 3,220 g and the aqueous phase was removed. After addition of four volumes of methanol, the proteins were pelleted via centrifugation for 10 min at 3,220 g. Pellets were washed with 80% acetone, and centrifuged for 10 min at room temperature at 3,220 g. The supernatants were discarded and the pellets were dried on air. Protein pellets were then resuspended in 8 M urea in 50 mM triethylammonium bicarbonate (TEAB) buffer (pH 8). Alkylation of cysteines was carried out by adding tris(carboxyethyl)phosphine (TCEP, Pierce™) and iodoacetamide (SigmaAldrich) to final concentrations of 15 mM and 30 mM, respectively, and the samples were incubated for 15 min at 30 °C in the dark under rotation. Next, the mixtures were diluted 8-fold with 50 mM TEAB, followed by an overnight digestion (37 °C) with Trypsin/Lys-C (Promega) with an enzyme-to-substrate ratio of 1:100 (w:w). The digest was acidified to pH 3 with trifluoroacetic acid (TFA) and desalted using SampliQ C18 SPE cartridges (Agilent) according to the manufacturer’s guidelines. The desalted peptides were fully dried in a vacuum centrifuge and then resuspended in 500 μl of loading solvent (80% (v/v) acetonitrile (ACN), 6% (v/v) TFA). Finally, 20 µl of the resuspended peptides were vacuum dried and retained for proteomics analysis. Samples were stored at -20 °C until LC-MS/MS analysis.

Peptides were re-dissolved in 20 µl solvent A (0.1% TFA in water/ACN (99.5:0.5, v/v)) of which 10 µl was loaded on an Ultimate 3000 ProFlow nanoLC system in-line connected to a Q Exactive HF mass spectrometer (Thermo Scientific™) equipped with a Nanospray Flex™ Ion Source (Thermo Scientific™). The peptides were separated on a 250-mm Odyssey Ultimate column with 1.7-µm C18-coated beads and a 75-µm inner diameter (Ionopticks) kept at a constant temperature of 45 °C. Peptides were eluted by a gradient reaching 26% MS solvent B (0.1% formic acid (FA) in ACN) after 75 min, 44% MS solvent B at 95 min, 56% MS solvent B at 100 min followed by a 15-min wash at 56% MS solvent B and re-equilibration with MS solvent A (0.1% FA). The flow rate was set to 250 nl/min. The mass spectrometer was operated in data-independent mode, automatically switching between MS and MS/MS acquisition. Full-scan MS spectra ranging from 375-1,500 m/z with a target value of 5E6, a maximum fill time of 50 ms, and a resolution at of 60,000 were followed by 30 quadrupole isolations with a precursor isolation width of 10 m/z for higher-energy collisional dissociation (HCD) at a normalized collision energy (NCE) of 30% after filling the trap at a target value of 3E6 for a maximum injection time of 45 ms. MS2 spectra were acquired at a resolution of 15,000 in the Orbitrap analyser. The isolation intervals ranging from 400–900 m/z, without overlap, were created with the Skyline software tool.

The mass spectrometry proteomics data have been deposited to the ProteomeXchange Consortium via the PRIDE partner repository with the dataset identifier PXD079829 (roots) and PXD078508 (leaves) (Perez-Riverol et al., 2025).

### 2.4 Database search, data filtering, and linear modeling

Raw files were searched using the DIA-NN software (version 1.8.1) with mainly default settings (Demichev et al., 2020). Spectra were searched in library-free mode against the *Glycine max* reference proteome sequence present in the UniProt database (March 2024). A precursor mass range filter of 400–900 m/z was applied, match between runs (MBR) option was enabled and “mass accuracy” (MS2 mass accuracy) and “MS1 accuracy” (MS1 mass accuracy) parameters were set at 20 ppm and 10 ppm, respectively. A maximum of one trypsin missed cleavage and of two variable modifications (acetylation on protein N-termini or oxidation of methionine residues) was allowed. Cysteine carbamidomethylation was enabled as a fixed modification.

First, the DIA-NN main report output table was filtered in RStudio (R version 4.4.0) at a precursor and protein library q-value cut-off of 1%, and only proteotypic peptides were retained. The report was then reshaped from long to wide format using the sample identifier as columns and protein group intensities (PG.MaxLFQ) as values. For proteins quantified multiple times per sample, intensities were averaged to obtain a single value per protein per sample. Next, LFQ-normalized protein intensities were log_2_-transformed in Perseus (versions 1.6.5.0 and 2.0.11.0) (Tyanova et al., 2016). Only proteins with valid quantification values in two technical replicates for at least one condition were retained. Imputation by random sampling from a normal distribution around the detection limit per sample (width = 0.3 and down shift = 1.8) was applied to assign missing values. A principal component analysis (PCA) was performed to investigate the sample distances, treating all quantified proteins in each sample as one datapoint.

Subsequently, the different conditions were compared using linear models implemented in the limma package, with statistical significance for differential regulation set at a false discovery rate (FDR) threshold of 0.05 (Ritchie et al., 2015). For the root proteomics dataset, a design matrix containing the factor “timepoint” was fitted, with replicate included as a blocking factor, followed by pairwise contrasts between timepoints. For the leaf proteomics dataset, the design matrix included genotype and timepoint as the explanatory factors, with replicate included as a blocking factor, followed by the contrasts between timepoints per genotype and between genotypes per timepoint. Statistical significance was determined using empirical Bayes moderation and an FDR threshold of 0.05. Since the proteomics experiment consisted of three technical replicates derived from a single pooled biological sample per condition and timepoint, the resulting statistical inference reflects technical variation only and does not capture biological variation between independent experiments.

Overlap of differential proteins between the conditions was analyzed using the VennDiagram and VennDetail packages (Chen and Boutros, 2011).

For the root proteomics analysis, to investigate further potential functional relationships between proteins, a protein–protein interaction (PPI) network was constructed using the STRING database, with a medium confidence (0.400) and including no more than 10 interactors as the maximum number of interactors shown. Extra filtering was done to exclude interacting proteins that had only been identified via text mining (Szklarczyk et al., 2023). The resulting network including predicted interactors was imported into Cytoscape (version 3.10.3) for visualization and functional analysis (Shannon et al., 2003). The MCODE plugin (version 2.0.0) was applied to identify densely connected clusters, potentially representing co-functional protein complexes or pathways, while CytoHubba was used to detect hub or bottleneck proteins that might serve as key regulators (Bader and Hogue, 2003; Chin et al., 2014). Functional enrichment analysis was performed for each identified cluster using the STRING plugin.

### 2.5 Heatmap construction

A heatmap was generated in RStudio (R version 4.4.0) using the pheatmap package (version 1.0.12) to visualize the patterns of differentially abundant proteins in the root proteome. The input data consisted of a z-score normalized matrix exported from Perseus (version 2.0.11.0), representing log_2_-transformed protein intensities across experimental conditions. Rows correspond to individual proteins, and columns to different timepoints and replicates. The heatmap was constructed using default clustering parameters (hierarchical clustering with Euclidean distance and complete linkage). Row and column dendrograms were included to highlight patterns of co-regulation and sample similarity. Proteins not retained after data filtering were excluded prior to visualization.

### 2.6 Phylogenetic tree construction

Nine Bet v I domain-containing proteins were identified in this study. Their amino acid sequences were aligned in MEGA version 11 (Tamura et al., 2021), with the MUSCLE algorithm (default parameters) (Edgar, 2004), and used as input for an HMMsearch against the *Glycine max* proteome (retrieved from UniProt, June 2025) (Finn et al., 2011; The UniProt Consortium, 2025). Protein variants originating from the same gene were removed and 41 protein sequences remained. Multiple sequence alignment was generated in MEGA version 11 with the MUSCLE algorithm. Subsequently, a phylogenetic tree was constructed by maximum likelihood inference, Jones-Taylor-Thornton (JTT) model (Gamma distributed with invariant sites), with 1,000 bootstrap replications (Felsenstein, 1981; Jones et al., 1992).

### 2.7 Metabolomic analysis of soybean leaves

Wild type Wm82 and *nod4* mutant leaves were harvested at 0 and 7 dpi. Leaves of six plants were pooled, frozen immediately in liquid nitrogen, ground into a fine powder and separated in six technical replicates for metabolite extraction. For each replicate, 1 ml of UPLC-grade methanol was added to 100 mg of sample. The samples were shaken for 30 min at room temperature and subsequently centrifuged for 3 min at 21,300 g. The upper phase, containing the methanol extract, was vacuum-dried. The remaining pellet was also dried and the weight of the pellet was used for normalization of the metabolite data.

Sample cleanup was performed using an Oasis HLB LP 96-well SPE plate (60-µm particle size, 60 mg sorbent; Waters^®^). Positive pressure was applied using an Otto SPEcialist system (Waters^®^) for 2 min at a maximum pressure of 4 psi. Prior to sample loading, the SPE plate was conditioned with 1 ml of UPLC-grade methanol. Following conditioning, the reconstituted samples were transferred to the wells and passed through the sorbent bed under positive pressure using the Otto SPEcialist. Each well was then washed with 100 µl of UPLC-grade methanol. The eluates and wash fractions were combined and transferred to 1.5-ml Eppendorf tubes for drying under vacuum. Dried samples were reconstituted by adding 20 µl UHPLC–MS-grade ACN and 80 µL UHPLC-MS-grade water. Samples were then centrifuged at 20,800 g, 8 °C, for 10 min to precipitate any insoluble material, and subsequently transferred into vials. To generate the pooled samples, 5 µl was taken from each sample and pooled.

Samples were injected into an Acquity UPLC equipped with a BEH C18 column (2.1 mm × 150 mm, 1.7 μm, Waters^®^) coupled to a Synapt Q-Tof (Waters^®^). A gradient of two buffers was used: buffer A (0.1% FA in water, pH 3) and buffer B (0.1% FA in ACN, pH 3); 99% buffer A was decreased to 0% buffer A in 30 min (350 μl/min, column temperature 40 °C). The flow was diverted to the mass spectrometer equipped with an electrospray ionization source and lockspray interface for accurate mass measurements. The source parameters were as follows: capillary voltage, 1.5 kV for ESI+ and 2.2 kV for ESI-; sampling cone, 40 V; extraction cone, 4 V; source temperature, 120 °C; desolvation temperature, 550 °C; cone gas flow, 50 l/h; and desolvation gas flow, 800 l/h. The collision energy for the trap and transfer cells was 4 V and 2 V, respectively. Full-scan data were recorded in both positive and negative profile modes; the mass range was from m/z 50 to 1,500, with a scan time of 0.2 s, using MassLynx v.4.2 software (Waters^®^). Leucine-enkephalin (100 pg/μl in water:ACN 1:1 (v/v), with 0.1% FA) was used for the lock-mass calibration, scanning every 180 s with a scan time of 0.2 s.

Metabolites were annotated based on internal and public databases: PhytoComp (in-house), NIST2020 (http://chemdata.nist.gov), Massbank (Horai et al., 2010), GNPS (Wang et al., 2016), ReSpect (Sawada et al., 2012), FoodDB (www.foodb.ca), PlantCyc (https://plantcyc.org/), ChEBI (Malik et al., 2026), Natural Product Atlas (Poynton et al., 2025), NANPDB (Ntie-Kang et al., 2017), COCONUT (https://coconut.naturalproducts.net/), and KNApSAcK (Afendi et al., 2012).

The metabolomics data have been deposited to MetaboLights repository with the study identifier MTBLS14770 (Yurekten et al., 2024).

During preprocessing of the data, features with a constant value across samples or having infinity values were removed. Missing data were imputed by replacing missing values and zero values by the lowest value detected. The data were arch-sinh (inverse hyperbolic sine) transformed and Pareto scaled (mean-centered and divided by the square root of the standard deviation of each variable). Then, the data were filtered to only retain features with a mean intensity parameter > -5. Subsequently, statistical analysis of the data was performed with a two-way ANOVA, significance was determined with the following threshold: adjusted P value < 0.01. All flavonoid compounds were selected based on the ontology and the normalized intensities were represented in a heatmap using the pheatmap package in RStudio as described above.

## 3 Results

### 3.1 Experimental set-up and timing of AON signaling

To select timepoints for analysis, we grew plants in an aeroponics system and sampled the roots and leaves at several timepoints between 0 and 14 dpi to analyze the onset of the AON pathway based on expression of 6 known marker genes *RIC1/2, miR2111c/f*, and *TML1a/1b*. The variability between the four replicate experiments that we performed was rather high (**Supplementary Figure S1**; **Supplementary Dataset S1**), probably because of biological variability between the independent plant–microbe interactions and because large root regions were isolated for RNA extraction. However, similar trends were observed. In the roots of WT Wm82 soybean, *RIC1* gene expression was increased at around 1–3 dpi, followed by fluctuations in gene expression and a decrease at 14 dpi. For *RIC2*, gene expression was induced later, between 5 and 7 dpi, and increased until 10 dpi. Next, gene expression of two *premiR2111* isoforms, *premiR2111c* and *premiR2111f*, was measured in soybean leaves. Overall, we observed a transient upregulation followed by a downregulation starting between 3 and 4 dpi for both isoforms. Finally, the expression of *TML1a* and *TML1b* in the roots decreased initially, followed by an increase starting at 5 dpi. This timing roughly coincided with the decrease of the *premiR2111* transcripts, which are described to target *TML* transcripts in the roots for degradation (Zhang et al., 2021). Significant differences were observed only for *RIC2*, increasing significantly between 0 and 10 and 0 and 14 dpi (P = 0.035 and P = 0.016, respectively), and for *premiR2111c*, decreasing significantly between 1 and 10 dpi and between 4 and 10 dpi (P = 0.037 and P = 0.007, respectively).

To study the proteome changes following nodulation in soybean, we selected three timepoints for shotgun proteomics based on these RT-qPCR data: 0, 3, and 7 dpi (**Figure 1**). Although the biological variation was high, similar overall temporal trends were observed across experiments, consistent with the expected AON signaling events, allowing the identification of 3 dpi and 7 dpi as biologically relevant timepoints for the subsequent proteomic analyses. Sampling at these timepoints was expected to give insights into the proteome before inoculation, and at early and late signaling. The *nark* mutant *nod4* was also included to explore NARK-dependent changes in the leaves. Gene expression analysis of the same AON marker genes confirmed activation of the AON signaling pathway in the newly harvested tissues (**Supplementary Figure S2**). Next, the samples were processed for the proteomics analysis to identify differentially abundant proteins in local and systemic signaling during nodulation. Tissues from 5–7 plants were pooled per condition to obtain a representative sample of the nodulation response. Each pooled sample was subsequently analyzed in three technical replicates.

**Figure 1:**
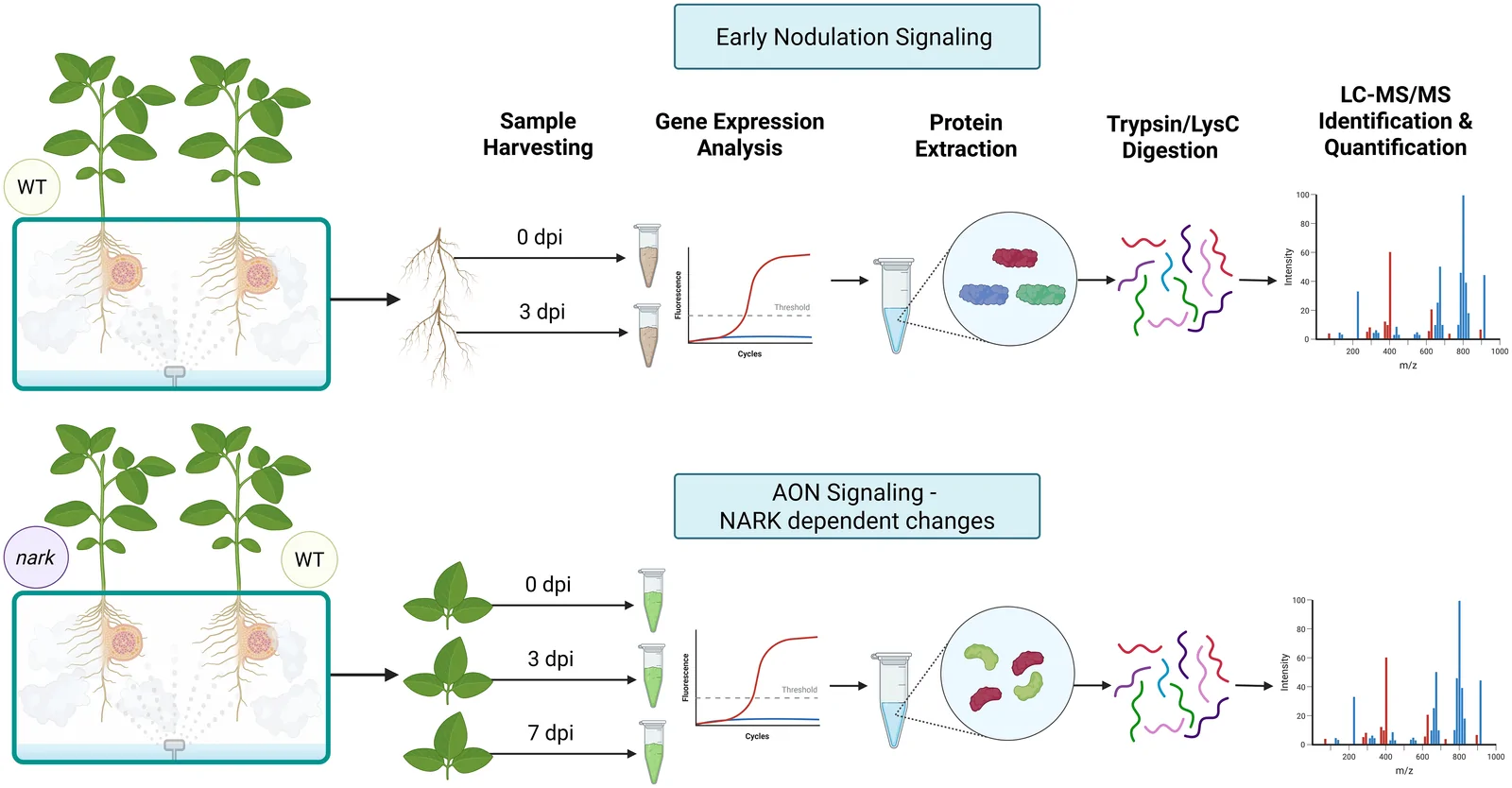
Experimental setup. Wild type (Williams 82, WT) soybean plants and nark mutant nod4 plants were grown in an aeroponics system and inoculated with *B. diazoefficiens*. In this system, soybean roots are continuously misted with Soli nutrient solution. (Above) To assess the early nodulation signaling changes (local signaling) at the protein level, WT root samples were harvested at 0 and 3 days post inoculation (dpi) and used for RNA extraction and gene expression analysis via RT-qPCR. Then, tissues from 5–7 plants were pooled per condition, used for total protein extraction, trypsin digestion, and LC-MS/MS analysis. Each pooled sample was subsequently divided into three technical replicates for proteomic analysis. (Below) Similarly, to assess the AON signaling and NARK-dependent changes (systemic signaling), WT and nod4 leaf samples were harvested at 0, 3, and 7 dpi and used for RNA extraction and gene expression analysis via RT-qPCR. Then tissues from 5–7 plants were pooled per condition, used for total protein extraction, trypsin digestion, and LC-MS/MS analysis. Each pooled sample was subsequently divided into three technical replicates for proteomic analysis. Created in BioRender.com.

For the roots, only the WT (Wm82) was included. The 7 dpi root samples were initially included in the analysis. During the initial analysis and prior to imputation, missing values were generally limited in the root proteomics dataset. However, missingness was disproportionately concentrated in the 7 dpi samples, particularly in one replicate (8.6% missing values compared with <2% for the other root samples in the original dataset including 7 dpi). As a result, inclusion of the 7 dpi condition required substantially more imputation than the 0 and 3 dpi samples and it would have affected a too large fraction of the overall dataset, which would have led to an artificial inflated number of significant proteins (data not shown). To ensure a robust biological interpretation of our proteomics data, the 7 dpi root samples were excluded from analysis (**Figure 2**), and the root proteomics dataset was focused on early stages when nodule organogenesis takes place and at the same time AON is activated. For the leaves, the *nark* mutant *nod4* was compared to the WT in order to identify AON-specific changes.

**Figure 2:**
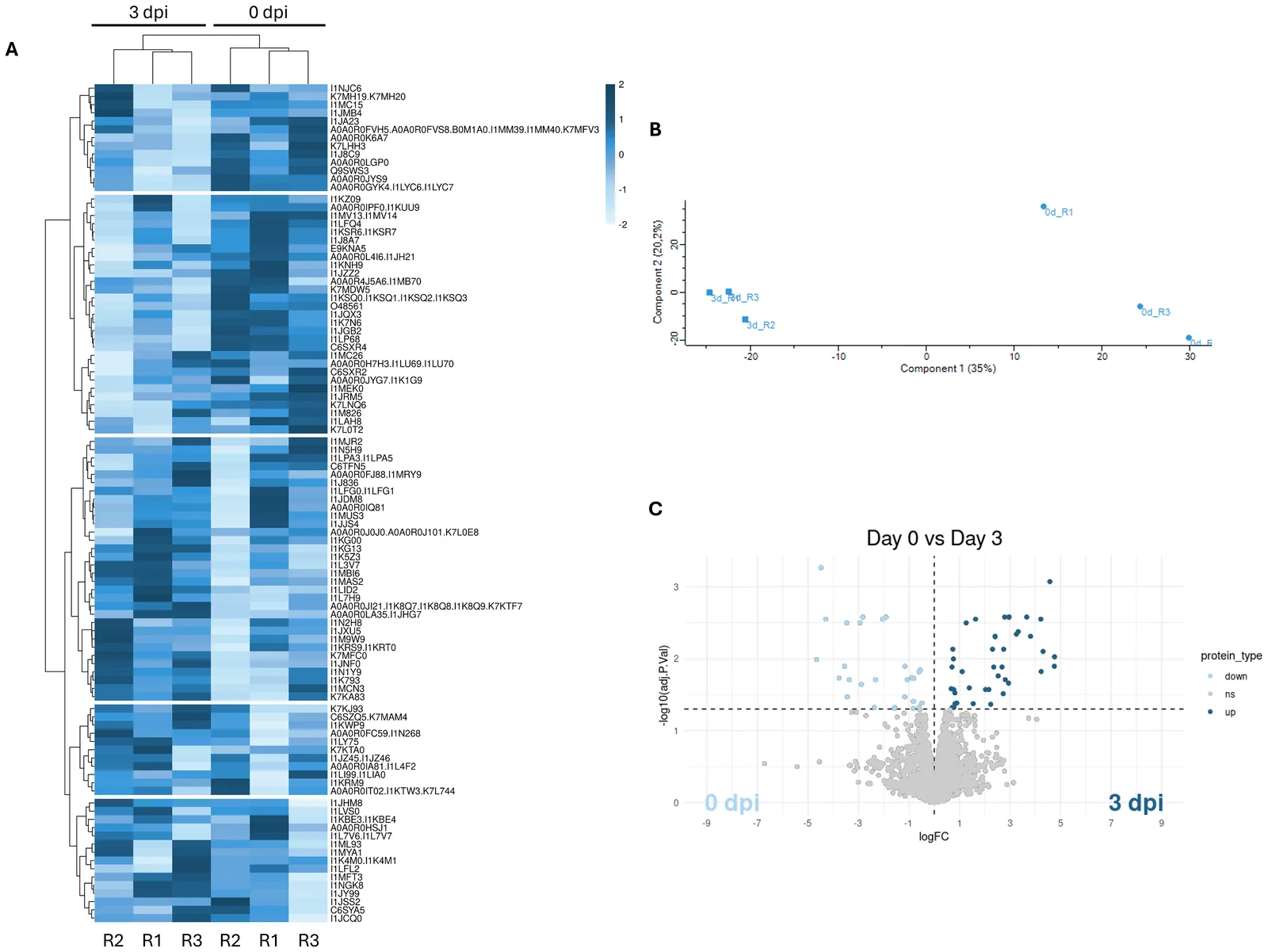
Proteome trends in soybean roots during early nodulation. **(A)** Protein abundance changes in Wm82 roots 3 days post-inoculation (dpi) with rhizobia compared to non-inoculated Wm82 roots. Heatmap showing protein abundance trends comparison between non-inoculated Wm82 roots and Wm82 roots at 3 dpi. Dark blue: higher abundance, light blue: lower abundance, for all panels. Values represent three technical replicates derived from a single pooled biological sample (5–7 plants) per condition. **(B)** Principal component analysis (PCA) plot of the shotgun proteomics data of Wm82 root samples (3 technical repeats). Samples cluster per timepoint. Circle: 0 dpi, square: 3 dpi. **(C)** Volcano plot showing proteome changes between non-inoculated Wm82 roots and Wm82 roots at 3 dpi. Black dashed lines indicate significance thresholds.

The raw MS spectra were analyzed by DIA-NN and yielded a total number of 20,315 and 21,016 unique protein identifications in roots and leaves, respectively. Subsequent data quality control and filtering was performed, followed by log_2_-transformation and data imputation. Prior to imputation, between 6,462 and 6,659 proteins were quantified per root sample and between 5,870 and 6,299 proteins per leaf sample, corresponding to approximately 4-7% and 7-13% missing values, respectively. Following imputation, 6,918 root proteins and 6,743 leaf proteins were available for downstream analyses. PCA revealed a clear clustering of root samples by timepoint, indicating distinct protein abundance trends between non-inoculated roots and those undergoing early nodulation (**Figures 2A, B**). In contrast, for the leaves, PCA analysis revealed that the samples clustered per replicate rather than per genotype or timepoint (**Supplementary Figure S3**). Since the samples from replicate 3 clustered separately from replicate 1 and 2, we decided to exclude this replicate from further analysis and focus on results from replicate 1 and 2 only. For completeness, we included the statistical analysis including all three replicates in Supplementary material (**Supplementary Dataset S5**). Some overlap was observed between both analyses, yet also unique significant proteins for each, however the proteins that are discussed below showed generally similar protein abundance dynamics in both analyses. Finally, 6,918 (roots) and 6,718 (leaves) protein identifications remained for statistical analysis by linear modeling using the limma package in RStudio.

### 3.2 Early nodulation: proteome and network analysis

To obtain an overview of proteomic changes during early nodulation in soybean roots, protein abundances were analyzed at 0 and 3 dpi. In total, 68 proteins differed significantly in abundance, with 41 significantly increased and 27 significantly decreased between 0 and 3 dpi (**Figures 2A, C**; **Supplementary Dataset S2**).

No Gene Ontology (GO) terms were found to be significantly enriched possibly because the number of significant changes identified was moderate. We therefore examined previously reported nodulation-related signatures and constructed a PPI network using the STRING database (Szklarczyk et al., 2023), which was further analyzed in Cytoscape. The resulting network in roots comprised 26 connected proteins, including 16 identified in the root proteomics dataset and 10 additional proteins introduced by SPRING based on known or predicted interactors (**Supplementary Figure S4**; **Supplementary Table S3**). Clustering analysis in Cytoscape identified three groups of interacting proteins. These clusters pointed to functional connections between differentially abundant proteins that were not apparent from individual annotations alone. Functional annotation via the STRING database suggested that cluster 1 was linked to general cellular processes such as RNA processing and transcription-related functions, cluster 2 contained proteins connected to immune-related responses, and cluster 3 included enzymes associated with tryptophan metabolism and oxidative activity (**Supplementary Figure S4**; **Supplementary Table S4**–**S6**).

Since no GO terms were enriched for the root dataset, we continued with targeted examination of proteins previously reported to be involved in nodulation, hormone signaling, nutrient responses, and related processes in legumes.

### 3.3 Protein changes linked to early nodulation in infected roots

Upon closer examination of the proteins that were found to have differential abundances at 3 dpi, some could be directly linked to nodulation (**Table 1**; **Figure 3A**). One of the proteins with a significantly increased abundance was a homolog of the *M. truncatula* DMI3 protein (K7L881), suggesting a positive role in nodulation signaling (Rival et al., 2012; Yuan et al., 2022). Additionally, a Ras-related Rab7 GTPase (hereby Rab7, C6SWS6) was identified, which has been previously located to the soybean symbiosome and is involved in vesicle trafficking processes essential for symbiosome development and maintenance, thus potentially supporting nodule formation and function (Clarke et al., 2015). Another key finding was the SCAR protein (SCAR2, K7K3J6), a putative soybean homolog of *L. japonicus* SCARN, a legume-specific member of the SCAR/WAVE actin regulatory complex, found to have decreased levels at 3 dpi (Qiu et al., 2015).

**Table 1:** Proteins linked to early nodulation responses, nutrient allocation, and immunity that show significant protein abundance changes in Wm82 roots 3 dpi with rhizobia.

Protein Name	ProteinID	Gene code	LogFC	Roles (known and predicted)
Proteins linked to early nodulation responses				
DMI3 protein	K7L881	Glyma.08G227200	2.68	Nodule organogenesis
Calcium-dependent protein kinase (CDPK10)	I1KCN9	Glyma.06G189600	2.79	Calcium signaling
Ras-related Rab7 GTPase (Rab7)	C6SWS6	Glyma.07G004500	2.73	Symbiosome maintenance
4-coumarate-CoA ligase-like 9 (4CL9)	I1N7E7	Glyma.19G075800	2.23	Isoflavone biosynthesis
Amidase domain-containing protein (AMI1)	I1NIY1	Glyma.20G232900	2.03	Auxin homeostasis
Lunapark domain-containing protein (LNP)	A0A0R4J3E4	Glyma.06G075600	-2.83	Root hair structure
SCAR protein (SCAR2)	K7K3J6	Glyma.01G128700	-2.33	Infection
Annexin (ANXA13)	A0A0R0GR41	Glyma.13G200000	-1.16	Nodulation
ABC transporter B family member 9 (ABCB9)	A0A0R0G992	Glyma.15G090200	-0.83	Auxin transport
Proteins linked to nutrient allocation				
Phosphoribosyltransferase domain-containing protein (PRS4)	I1J550	Glyma.01G026800	2.36	Nucleotide and amino acid synthesis
Diacylglycerol O-acyltransferase 2D (DGAT2D)	K7K424	Glyma.01G156000	2.97	Triacylglycerol (TAG) synthesis
Major facilitator superfamily (MFS) profile domain-containing protein (NRT2.4)	A0A0R0HJF2	Glyma.11G195200	2.41	Nitrate transport
Tyrosine-specific protein phosphatase (DSP4)	I1NHQ7	Glyma.20G190400	0.73	Starch degradation
CBS domain-containing protein (KING1)	A0A0R0FNH1	Glyma.17G129600	-4.48	Sugar signaling
SIS domain-containing protein (PHI-like)	I1NGR9	Glyma.20G157100	-2.89	Sugar signaling
Proteins linked to immunity				
Transportin MOS14	A0A0R0FLG5	Glyma.17G094800	4.22	Splicing of Resistance genes
Bet v I/Major latex domain-containing protein (Bet15)	C6SYE7	Glyma.15G217100	0.82	Plant defense and development

**Figure 3:**
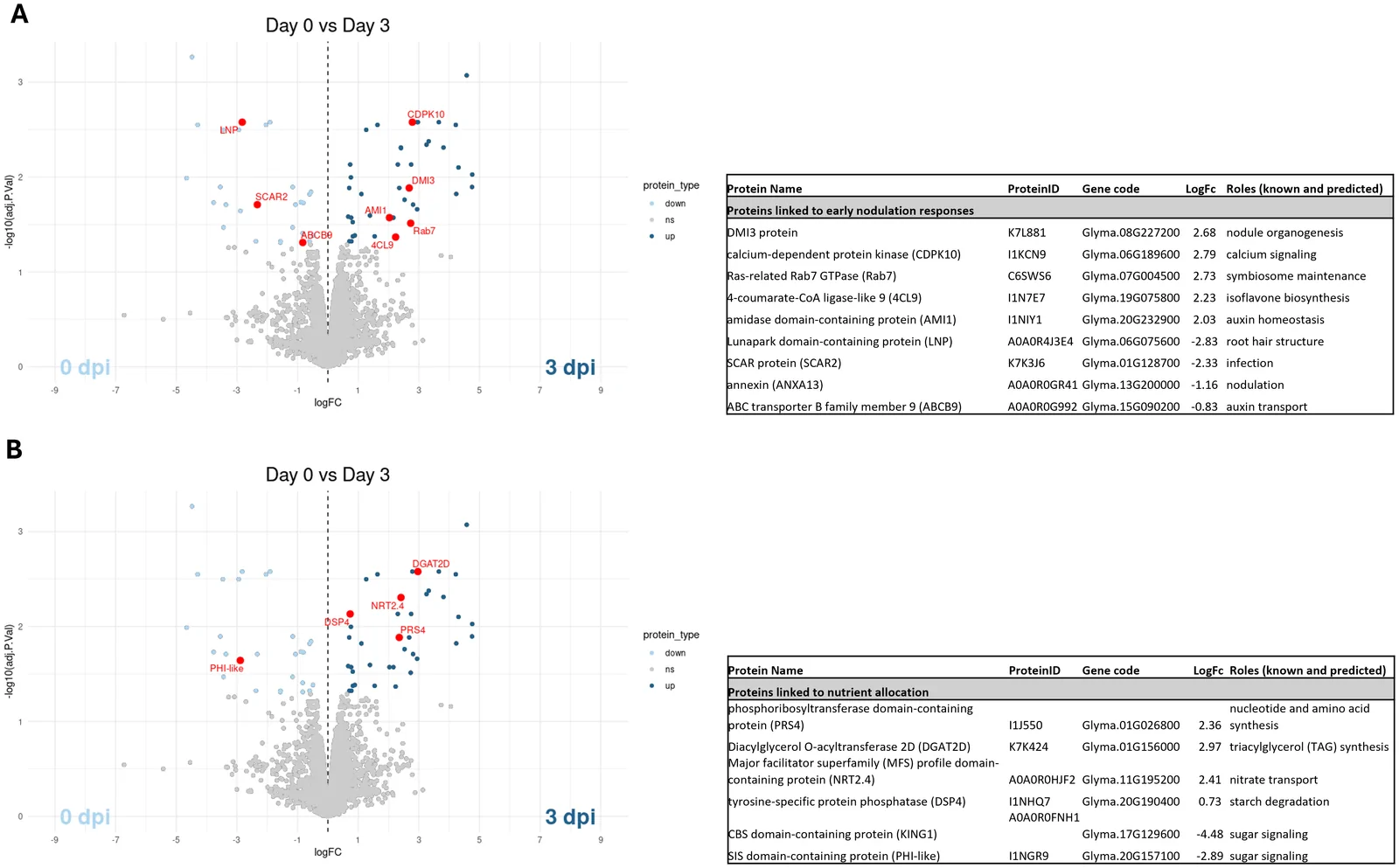
Protein abundance changes in Wm82 roots 3 days post-inoculation (dpi) with rhizobia compared to non-inoculated Wm82 roots. Volcano plots showing proteome changes between non-inoculated Wm82 roots and Wm82 roots at 3 dpi. Black dashed lines indicate significance thresholds. Light blue dots are proteins with significantly decreased abundance, dark blue dots proteins with significantly increased abundance, gray dots are non-significant (ns) changes. Red dots indicate proteins of interest (related to nodulation signaling, nutrient allocation, and immunity). **(A, B)** Volcano plots indicating proteins with links to early nodulation signaling **(A)** or nutrient allocation **(B)**; the soybean gene codes and protein descriptions and functions are provided in the tables at the right.

Additionally, several proteins enriched or depleted in nodulated roots could be indirectly linked to known nodulation processes. A calcium-dependent protein kinase (CDPK10, I1KCN9), which might be associated to the activation of calcium signaling during nodulation, was found with increased abundance. Proteins associated with cellular reorganization were also identified, including the Lunapark domain-containing protein (LNP, A0A0R4J3E4), whose *A. thaliana* homolog has been implicated in ER remodeling during root hair development, and annexin A13 (ANXA13, A0A0R0GR41), homologous to annexins characterized in *M. truncatula* and *A. thaliana*, which have been implicated in membrane-associated calcium signaling and early nodulation responses, as shown for *MtAnn1* (Perrine-Walker et al., 2014; Guillory et al., 2024; Upadhyay et al., 2025; Xiao et al., 2025).

Finally, two proteins associated with auxin regulation were identified. AMI1 (I1NIY1), involved in auxin biosynthesis, showed an increased abundance at 3 dpi. In contrast, the auxin transporter ABCB9 (A0A0R0G992) was less abundant.

### 3.4 Protein changes linked to nutrient allocation in infected roots

Among the proteins with differential abundance at 3 dpi in Wm82 roots, several exhibited functions indirectly associated with nutrient allocation or metabolic rewiring (**Table 1**; **Figure 3B**). More specifically, a phosphoribosyltransferase domain-containing protein (PRS4, I1J550) was identified, putatively functioning as a ribose-phosphate pyrophosphokinase (PRPP synthetase). Its *A. thaliana* homolog, AtPRS4, catalyzes the synthesis of phosphoribosyl pyrophosphate, a key precursor in nucleotide, amino acid, and cofactor biosynthesis (Busche et al., 2021). Additionally, a diacylglycerol O-acyltransferase 2D (DGAT2D, K7K424), was found to have significantly increased levels (Chen et al., 2016). A SIS domain-containing protein found to have lower abundance at 3 dpi was annotated as a 3-hexulose-6-phosphate isomerase-like (PHI-like, I1NGR9). This could reflect a potential shift in sugar flux, possibly to allocate energy to infection sites in the early stages of symbiosis.

Several other proteins were putatively associated with SnRK1 signaling, potentially linking the observed proteomic changes to carbon allocation and energy signaling. One such protein is the tyrosine-specific protein phosphatase (DSP4, I1NHQ7), a protein homologous to *A. thaliana* SEX4 (AT3G52180), a dual-specificity phosphatase involved in starch degradation. In *A. thaliana*, SEX4 dephosphorylates glucosyl residues on starch granules, facilitating carbon mobilization (Sokolov et al., 2006). It is also predicted *in silico* to interact with AKIN11, a functionally redundant kinase to AKIN10, which is the primary catalytic subunit of the SnRK1 complex in *A. thaliana*. Additionally, a CBS domain-containing protein (KING1, A0A0R0FNH1) displayed decreased abundance at 3 dpi, homologous to AT3G48530 in *A. thaliana* and annotated as an SNF1-related protein kinase regulatory subunit gamma 1. While not part of the canonical SnRK1 complex in plants, the *A. thaliana* homolog was reported to interact with HEXOKINASE 1 (HXK1), a key regulator of sugar sensing and carbon allocation (Emanuelle et al., 2015; Van Dingenen et al., 2019; Van Leene et al., 2022).

Finally, a major facilitator superfamily (MFS) domain-containing protein (NRT2.4, A0A0R0HJF2), annotated in soybean as “high-affinity nitrate transporter 2.4,” was identified as more abundant at 3 dpi. Homologous to *A. thaliana* NRT2.4 (AT5G60770), and supported by KEGG and Panther annotations linking it to the NRT2 family, this protein is predicted to function in high-affinity nitrate uptake and translocation from roots to shoots, suggesting a possible role in nitrogen acquisition during early symbiosis (Kiba et al., 2012).

### 3.5 From local signaling in the roots to systemic control of nodulation in the leaves

As nodulation continues and biological nitrogen fixation is performed, the plant needs to balance its resources and move from local signaling (root) to systemic control (AON) via the leaves to achieve optimal symbiosis regulation. A central player in systemic AON signaling is NARK, which is why *nark* mutants (*nod4*) were included for leaf proteomics analysis.

For the WT, Wm82, significant abundance changes were observed for 25, 5 and 45 proteins between 0-3, 3 -7 and 0 -7 dpi respectively (**Figures 4A–C**; **Supplementary Dataset S3**). Early changes were associated with oxylipin biosynthesis and oxidoreductase activity (increased) and DNA/RNA processing and immunity (decreased). In the *nod4* mutant, more protein abundance changes were observed, respectively 32, 46 and 142 between 0 -3 dpi, 3 -7 dpi and 0 -7 dpi, with mainly increased abundances (**Figures 4D–F**). Among the proteins with increased abundance, six lipoxygenases were detected, of which three also showed significantly increased abundance in Wm82. The changes covered a broad range of GO terms, while also the number of proteins that belonged to every category was limited. Since GO analysis did not yield many insights, we decided to focus mainly on two interesting features that were present in the dataset: immunity-related proteins and isoflavonoid biosynthesis.

**Figure 4:**
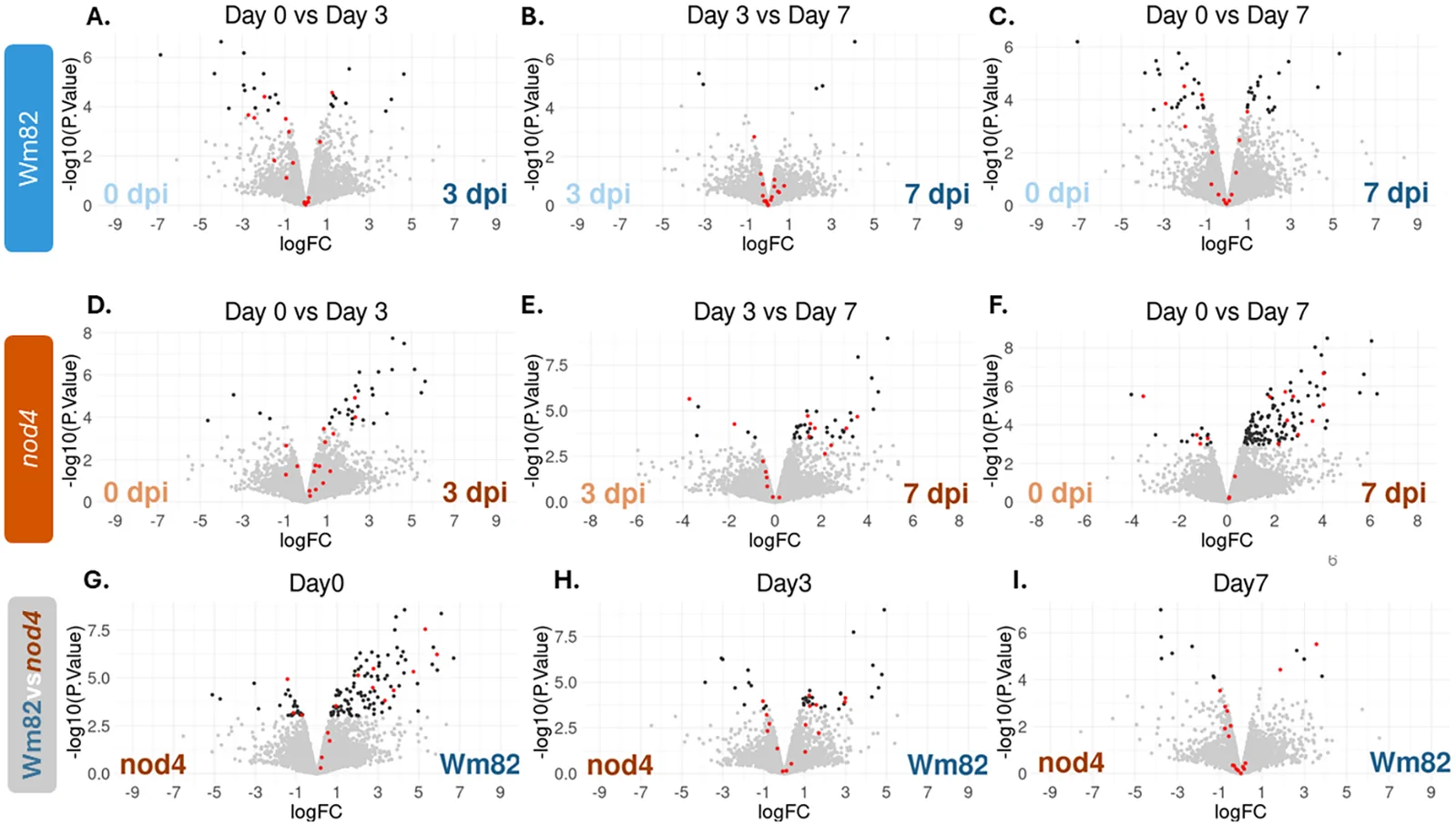
Volcano plots depicting the proteome changes related to immunity in Wm82 and *nod4* leaves. **(A–E)** Volcano plots for timepoint comparisons 0 dpi versus 3 dpi b **(A, D)**, 3 dpi versus 7 dpi **(B, E)** and 0 dpi versus 7 dpi **(C, F)**. **(A–C)** Volcano plots for Wm82. **(D–F)** Volcano plots for *nod4*. **(G–I)** Volcano plots depicting the differential protein abundances in Wm82 versus *nod4* at 0 dpi **(G)**, at 3 dpi **(H)** and at 7 dpi **(I)**. Proteins related to immunity are indicated in red, proteins with significant protein abundance changes in dark gray, with non-significant changes in light gray.

Overall, most protein abundance changes were unique for either the WT Wm82 or the *nark* mutant *nod4*. The number of significant changes was larger in the *nark* mutant than in Wm82. Furthermore, fewer differences in protein abundances were observed between the two genotypes at the later timepoints; at 0 dpi the abundance of 131 proteins was significantly different in Wm82 compared to *nod4*, while at 3 dpi only 41 and at 7 dpi only 12 (**Figures 4G–I**). In short, the data indicate that the two genotypes differed in their basal state and that the proteome differences between them decreased over time following nodulation. Accordingly, the number of immunity-related proteins which had a significantly different abundance at 0 dpi in Wm82 – most of them had a higher abundance in Wm82 – dropped over time.

### 3.6 Differential immunity responses in Wm82 and *nod4* mutant leaves

Interestingly, we observed that some proteins with a putative link to immunity were affected in the leaves (**Figure 5A**; **Supplementary Figure S5A**; **Supplementary Dataset S3**). Generally, lower protein abundances were observed in the *nod4* mutant at 0 dpi, followed by a significant increase until 7 dpi, when similar levels were reached as in Wm82. The protein levels did not significantly alter in Wm82 over time. The immunity-associated proteins included: plant basic secretory protein (BSP) family proteins (I1NFY4, C6SYB8 and I1LEI5), chitinase (I1M587), pathogen-related protein-like proteins (PRPL, A0A0R0JPA5 and A0A0R0JFW9), MACPF-domain containing protein (I1M1M7), Gnk2-homologous domain containing proteins (A0A0R0HYZ6 and K7LLD6), pathogenesis-related protein 1 (PR-1, I1ME54), hypothetical enhanced disease susceptibility 1 (EDS1) homologue (I1KCL7), hypothetical C-terminally Encoded Peptide Receptor 2 (CEPR2, I1LNP8) homologue, and mitogen-activated protein kinase 4 (MKK4, A0A0R4J3M6) (**Supplementary Dataset S3**). For the CEPR2 and MKK4 proteins, a deviating abundance pattern was observed: the abundance was reduced in *nod4* leaves at 7 dpi. Interestingly, for the BSP proteins opposite protein dynamics were observed in Wm82 leaves (reduced abundance) compared to *nod4* (increased abundance).

**Figure 5:**
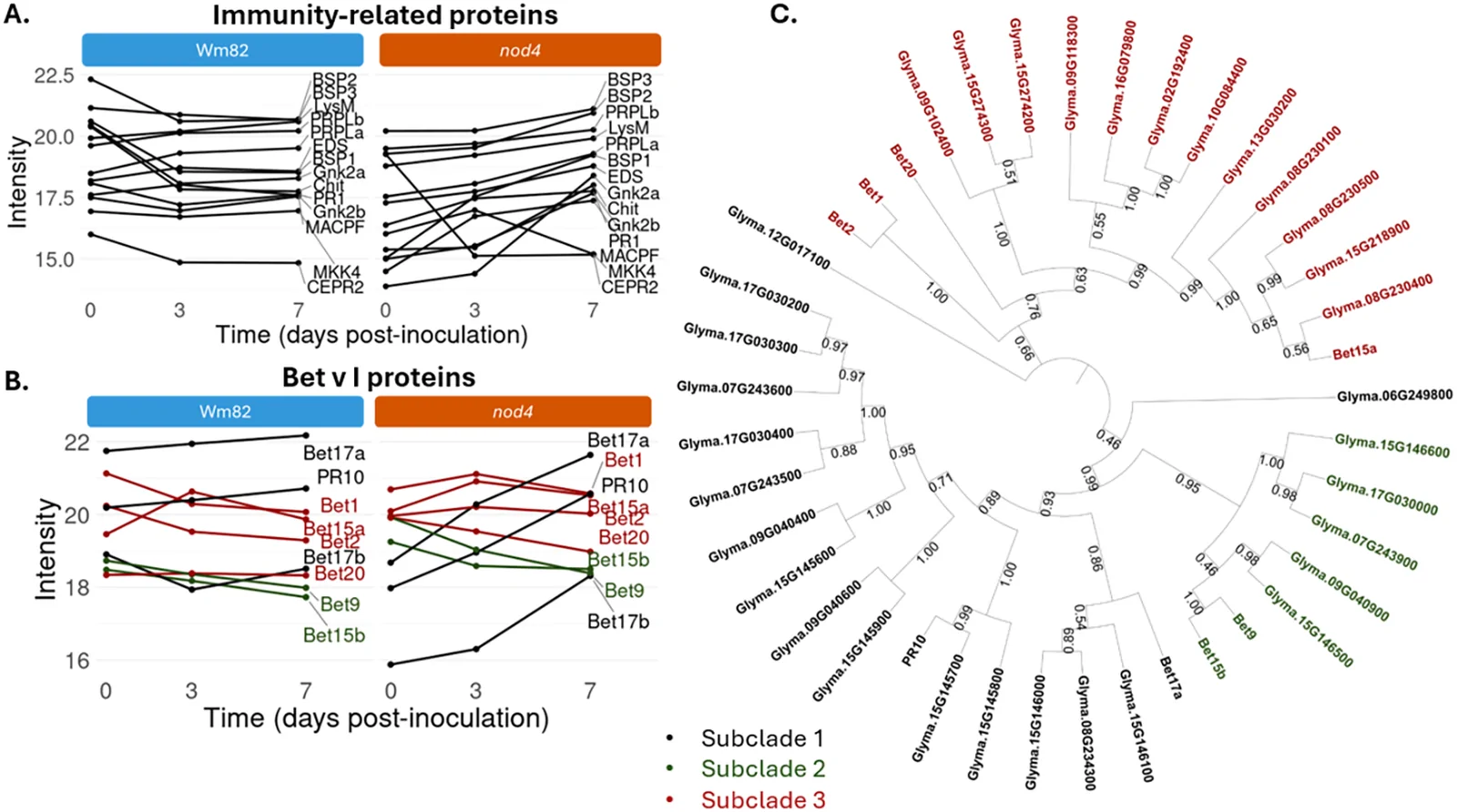
Protein profile plots of immunity-related proteins and phylogenetic tree of Bet v I domain-containing proteins. **(A)** Protein profile plot of immunity-related proteins in Wm82 and *nod4* leaves during nodulation. **(B)** Protein profile plot of Bet v I domain-containing proteins in Wm82 and *nod4* leaves during nodulation. Red, green and black lines represent Bet v I proteins from separate subclades, the colors correspond to the phylogenetic tree in panel **(C)** The log_2_ LFQ values (Intensity) of replicate 1 are represented in **(A, B)**, the profile plots with values from replicate 2 can be found in **Supplementary Figures S5A, B**. Protein names can be found in **Supplementary Dataset S3**. **(C)** Unrooted phylogenetic tree depicting Bet v I/Major latex domain-containing proteins in *Glycine max*. The alignment of the amino acid sequences of the Bet v I proteins identified in this study (**Supplementary Dataset S3**) was used as input for an HMM search, identifying additional Bet v I/Major latex-domain containing proteins in *Glycine* max. The amino acid sequences were downloaded from Uniprot (June 2025) (The UniProt Consortium, 2025) and aligned in MEGA version 11 (Tamura et al., 2021) with the MUSCLE algorithm (Edgar, 2004). The phylogenetic tree was constructed by the Maximum-Likelihood method with 1000 Bootstrap replications (bootstrap confidence values on the branches are expressed as a percentage of the 1000 bootstrap replications) (Felsenstein, 1981).

Furthermore, several proteins of the Bet v I protein family were identified in our data (**Figure 5B**, **Supplementary Figure S5B**; **Supplementary Dataset S3**). These proteins belong to family 10 of the pathogenesis-related proteins (PR-10) and have been associated with immunity-related processes, although their precise biological functions and the extent to which they contribute to immunity remain unclear (Wen et al., 1997; Radauer et al., 2008). A phylogenetic tree of soybean Bet v I proteins indicated that this protein family in soybean can be divided in three subclades, which were associated with distinct protein abundance patterns (**Figures 5B, C**). Bet17a, Bet17b, and PR10 showed increased protein abundances in *nod4* leaves. At 0 dpi, their abundances were lower compared to those in Wm82 and, finally, at 7 dpi similar levels in both genotypes were observed. Bet15b and Bet9 showed significantly lower protein levels in Wm82 compared to *nod4* at 0 dpi, with a tendency to decrease in *nod4* over time, yet only significant for Bet9. In contrast, the members from the third clade, Bet1, Bet2, Bet15a, and Bet20, showed significant but more limited changes in expression.

Two proteins previously associated with immunity-related processes were also identified with significantly increased abundances in the roots at 3 dpi (**Table 1**): Bet15a and transportin MOS14 (A0A0R0FLG5). The closest homolog of MOS14 in *A. thaliana* is MOS14 (AT1G12930), a transportin-SR (TRN-SR) involved in plant immunity and the proper splicing of resistance genes. Therefore, the soybean protein may be associated with similar immunity-related processes, although its function has not been experimentally demonstrated in soybean (Xu et al., 2011; Aglas et al., 2020).

### 3.7 Isoflavonoids accumulate differentially in Wm82 and *nod4* leaves

Several proteins involved in (iso)flavonoid biosynthesis showed increased abundances in *nod4* leaves and relatively stable protein levels in Wm82 leaves following rhizobia inoculation (**Figure 6A**; **Supplementary Dataset S3**). Prior to inoculation, the protein abundances were lower in *nod4*, while at 7 dpi they were similar in both genotypes. Enzyme activities were associated with the proteins identified here, based on their EC numbers and the KEGG database (Ogata et al., 1999; Van Bel et al., 2022). The identified proteins were predicted to be involved in different steps of the flavonoid and isoflavonoid biosynthesis pathways. Intriguingly, the 2-hydroxyisoflavanone dehydratase (HID), the isoflavone 4’-O-methyltransferase (I4OMT), the isoflavone reductase (IR), and the vestitone reductase (VR) are involved in subsequent steps of pterocarpan biosynthesis, a subclass of isoflavonoids (**Figures 6B, C**). More specifically, they catalyze subsequent steps for the conversion of isoflavanone via daidzein and formononetin to medicarpin or pisatin. HID and I4OMT can also be involved in the production of biochanin A from naringenin through a genistein intermediate. Additionally, the isoflavone 7-O-methyltransferase (I7OMT) can convert daidzein into isoformonetin and genistein into prunetin. Finally, the malonyltransferase MT6 can conjugate glycosylated forms of multiple intermediates of this pathway, including formononetin, daidzein, medicarpin, genistein, and biochanin A.

**Figure 6:**
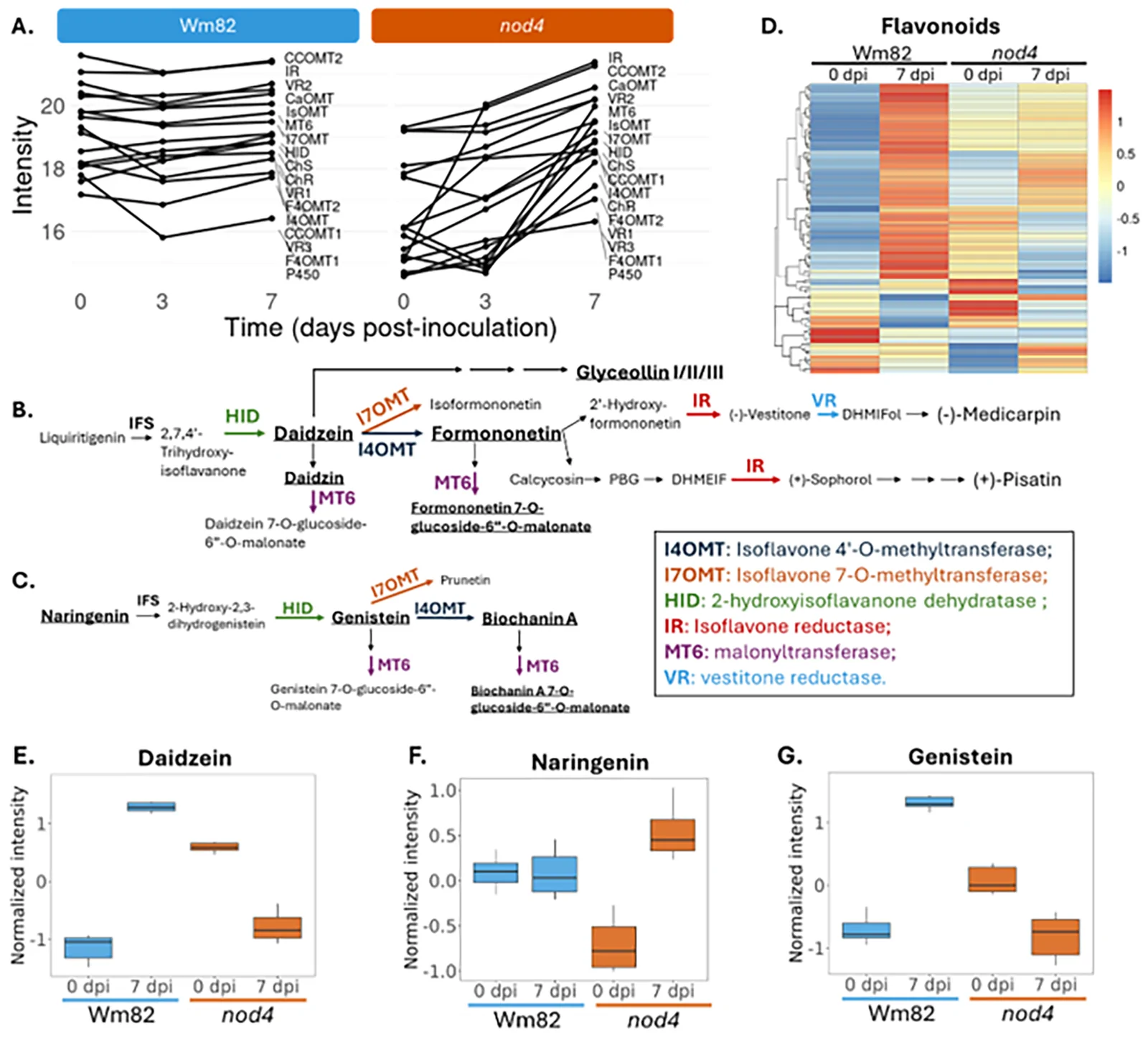
Isoflavonoid biosynthesis proteins, metabolic pathway and isoflavonoid accumulation in soybean leaves during nodulation. **(A)** Protein profile plot of (iso)flavonoid biosynthesis proteins in Wm82 and *nod4* leaves during nodulation. The log_2_ LFQ values (Intensity) of replicate 1 are represented, the profile plot with values from replicate 2 can be found in **Supplementary Figure S5C**. Protein names can be found in **Supplementary Dataset S3**. **(B)** Part of the pterocarpan biosynthesis pathway producing medicarpin and pisatin via daidzein and formononetin. **(C)** Metabolic pathway producing genistein and biochanin **(A)** The proteins that showed increased abundance in *nod4* leaves after inoculation with rhizobia are indicated with their abbreviation. They are associated with enzyme activities based on their EC number in the KEGG database (Ogata et al., 1999). DHMIFol: 7,2’-Dihydroxy-4’methoxy-isoflavanol, PBG: Pseudobaptigenin, DHMEIF: 2’,7-Dihydroxy-4’,5’-methylenedioxy-isoflavone. Metabolites in bold were detected by metabolomics (**Supplementary Dataset S4**). **(D)** Heatmap depicting the relative changes of flavonoid metabolite levels between the different samples (flavonoid metabolites are listed in **Supplementary Dataset S4**). Red: higher abundance, blue: lower abundance. **(E–G)** Boxplots representing the normalized intensities of selected compounds: **(E)** daidzein (m/z: 255.07, retention time (RT): 7.28), **(F)** naringenin (m/z: 273.08, RT: 8.27), and **(G)** genistein (m/z: 271.06, RT: 11.21 min).

To further investigate whether isoflavonoids were differentially regulated in Wm82 and *nod4*, we performed untargeted metabolomics. Leaves of both genotypes were analyzed at 0 and 7 dpi, and six technical replicates derived from a pooled sample were analyzed per condition. In general, the PCA plot indicated that the Wm82 and *nod4* samples at 0 dpi were more clearly separated, hence more different from each other, while they clustered more closely to each other at 7 dpi (**Supplementary Figure S6A**). The data indicate that the differences in metabolite levels of the leaves decreased over time during AON signaling.

In total, 8,874 metabolites were detected of which 2,045 showed a significantly different response over time depending on the genotype. Of these, 377 could be annotated using the MS-FINDER tool with the public and in-house libraries, which are listed in the Materials Section (**Supplementary Dataset S4**) (Tsugawa et al., 2016). The total scores that indicate the confidence of annotation can be found in **Supplementary Dataset S4**. Clearly, metabolite identification remains challenging in LC-MS-based untargeted metabolomics (Tsugawa et al., 2021; Giera et al., 2022). From these annotated (and significant) metabolites, 95 belonged to the flavonoids, including the isoflavonoid subclass (**Supplementary Dataset S4**). Most of the flavonoid metabolites showed increased accumulation in Wm82 at 7 dpi, while the changes in *nod4* leaves were more limited (**Figure 6D**). We focused in particular on the metabolites linked to the enzymatic activities that were identified before. Six intermediates of the pathways depicted in **Figures 6B, C**, were detected: daidzein, formononetin, glyceollin II, naringenin, genistein, and biochanin A (**Figures 6E–G**; **Supplementary Figures S6B–D**; **Supplementary Dataset S4**). Except for naringenin, a clear accumulation was observed in Wm82 between 0 dpi and 7 dpi, whereas in the *nod4* mutant, no changes or a slight decrease was detected. In particular, genistein and daidzein are well-known soybean metabolites.

For some metabolites, including formononetin and genistein, multiple fragments were detected with the same m/z, but different retention times, these represent different isomers which could not be distinguished with MS data. In-depth analysis is required to discriminate these structures (Schrimpe-Rutledge et al., 2016). However, they had similar abundance patterns, except for one genistein isomer (**Figure 6G**; **Supplementary Figure S7**). Similar abundance profiles were also observed for the 7-*O*-glucoside of daidzein (daidzin), the 3’-(gamma,gamma-dimethylallyl)genistein and the malonyl conjugates, formononetin 7-(6’’-malonylglucoside) and biochanin A 7-(6-malonylglucoside) (**Supplementary Dataset S4**). The malonyl isoflavones are important as storage forms; they are formed from the glycosylated conjugates, such as genistin or daidzin, and further converted by MT6 enzymes (**Figures 6B, C**) (Yang et al., 2022b).

Overall, during nodulation, isoflavonoid biosynthesis proteins had an increased abundance in *nod4* leaves, while flavonoid levels increased in the WT leaves. This apparent disconnect between enzyme abundance and metabolite accumulation suggests additional regulatory mechanisms acting downstream of protein accumulation and warrants further investigation.

Furthermore, jasmonic acid (JA) levels were detected at higher levels at 0 dpi in *nod4* leaves compared to Wm82 (**Supplementary Figure S6E**). A subsequent decrease in *nod4* leaves resulted in lower JA levels at 7 dpi in the mutant compared to the WT, where no significant changes were detected over time.

## 4 Discussion

In this study, we aimed at unraveling early nodulation and AON signaling players in soybean roots and leaves, respectively, by conducting a proteomics study. In the roots, we focused on the early stages of nodulation (0–3 dpi), while we included an additional later timepoint (7 dpi) for shoot tissues from the same plants to study systemic regulation of nodulation (**Figure 1**). For the shoot proteomics, WT and *nark* mutant leaves were compared in order to identify putative NARK-dependent changes. The parallel analysis of roots and leaves allowed to monitor both local and systemic responses to rhizobia, providing new insights into soybean nodulation.

To reduce variation associated with potential asynchronous nodulation and to increase the likelihood of detecting systemic responses, tissues from multiple plants (5-7) were pooled per condition prior to proteomic and metabolomic analysis, to obtain a representative sample of the nodulation response. Preliminary proteomics experiments (data not shown) and RT-qPCR analyses indicated substantial biological variation between individual plant-rhizobium interactions. This variation likely results from the asynchronous progression of nodulation events among individual plants, as well as the dilution of localized signaling responses when analyzing complete roots and leaves, since the biological activity presumably takes place in the vascular tissues (Okamoto et al., 2013; Zhang et al., 2021; Song et al., 2022). After pooling, the material was divided into three technical replicates for sample processing and LC-MS/MS analysis. These technical replicates were used as repeated measurements of the same pooled biological samples and were not considered independent biological replicates. Accordingly, the resulting statistical analyses reflect technical reproducibility rather than biological variation between individual plants. We hypothesized that this strategy would enable the detection of consistent molecular changes that might otherwise remain unidentified due to biological heterogeneity. Nevertheless, because independent biological replicates were not included, trends in the present datasets should be interpreted as representative trends within the experimental system and further evaluated in future studies.

In addition protein abundance changes can represent only one layer of regulation. Translation, post-translational modifications such as redox-dependent regulation, or protein-protein interactions can greatly affect protein activity without altering abundance (Vogel and Marcotte, 2012; Minguillón et al., 2022; Sang et al., 2025). Furthermore, intrinsically disordered protein regions and small signaling peptides, including the known AON-related CLE peptides, can provide additional levels of regulation that are not captured by the proteomics approach used here (Reid et al., 2011b; Sun et al., 2013; Frei dit Frey and Spallek, 2026).

### 4.1 Timing of AON signaling based on the expression of marker genes

The expression analysis of 6 marker genes of the AON signaling cascade confirmed the established sequence of AON signaling events. Although the expression was quite variable between different biological replicate experiments, the expression patterns of the AON marker genes were consistent with previous reports, showing early induction of *RIC1/2* in the roots, followed by downregulation of *miR2111* in the leaves and subsequent accumulation of *TML* transcripts in the roots to inhibit nodulation (**Figure 7B**) (Magori et al., 2009; Reid et al., 2011a; Tsikou et al., 2018; Zhang et al., 2021; Chaulagain et al., 2024). Our time series experiment was elaborate, comprising ten timepoints between 0 and 14 dpi. This allowed to analyze gene expression patterns in more detail, for example the downregulation of *RIC1* at later timepoints, in contrast to the continued upregulation of *RIC2*, which has not been described earlier. This corroborated the suggestion of both RIC peptides performing a slightly different function, which was proposed based on the later induction of *RIC2* compared to *RIC1* (Reid et al., 2011a). For the *premiR2111c/f* genes, an early upregulation was observed, before the previously described downregulation (Zhang et al., 2021). Downregulation was more clearly detected for the *premiR2111c* isomer. Similarly, for *TML1a/1b*, an early downregulation was observed, before transcript accumulation starting at 5 dpi. These results support the selection of 3 dpi as an early nodulation signaling stage and 7 dpi as a later stage associated with systemic AON responses for the subsequent proteomic analyses.

**Figure 7:**
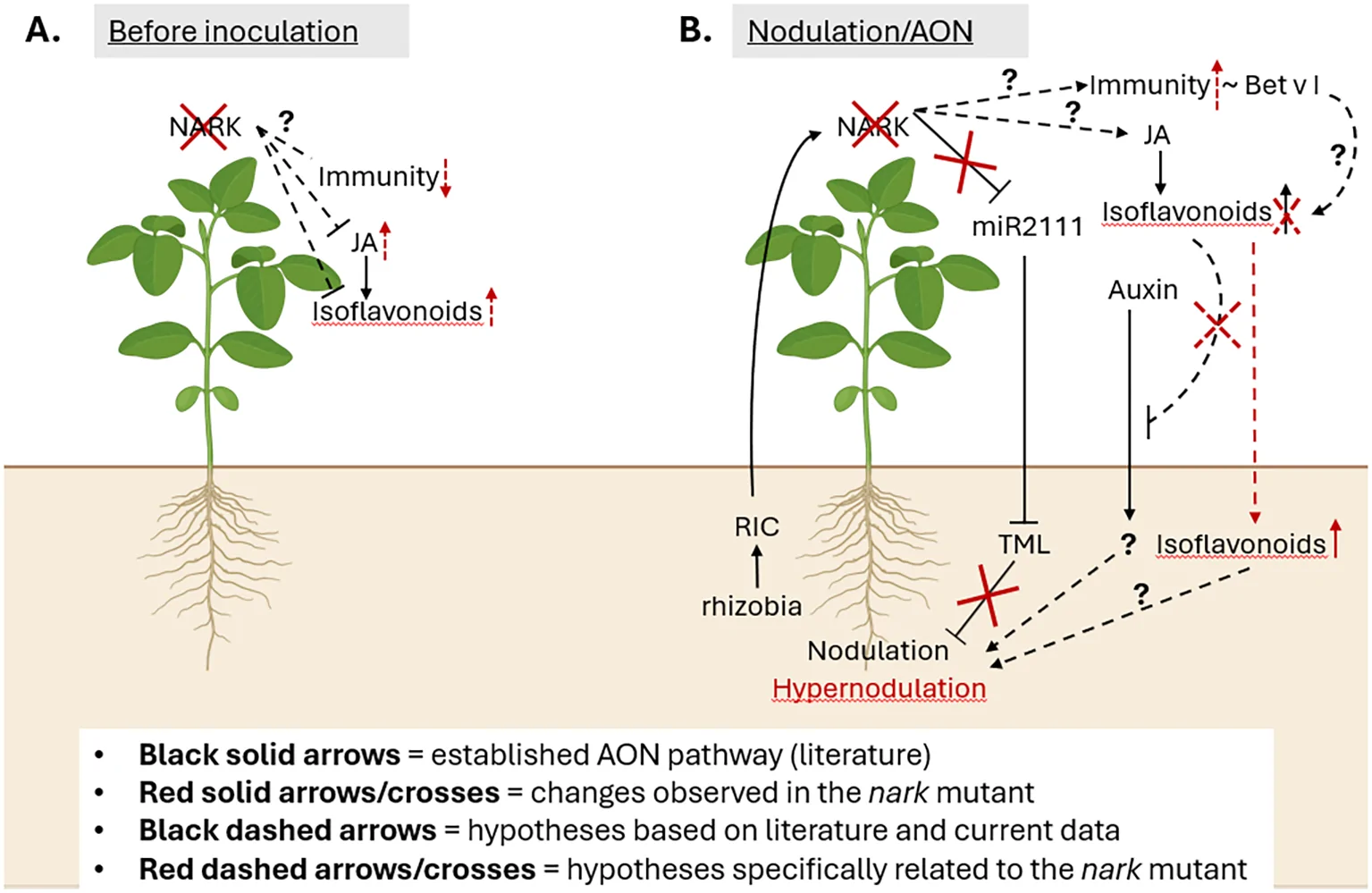
Overview figure summarizing the findings in this study regarding systemic signaling in the leaves. **(A)** In the absence of rhizobia, NARK may contribute to the regulation of jasmonic acid (JA) and isoflavonoid levels in leaves. In the *nark* mutant, the abundance of several immunity-associated proteins is reduced, while endogenous JA and isoflavonoid levels are elevated compared to the wild type. **(B)** In the presence of rhizobia, symbiotic nodulation is initiated and systemic AON signaling is activated. Rhizobia induce the production of RIC peptides in soybean roots, which are transported to the shoot, where they are perceived by NARK. Downstream signaling results in the downregulated shoot-to-root transport of miR2111, which allows TML accumulation in the roots, leading to inhibition of nodulation. Additionally, leaf isoflavonoid levels are significantly increased, which could lead to reduced shoot-to-root auxin transport. In *nark* mutants, AON signaling is disturbed, resulting in a hypernodulation phenotype. Immunity-related proteins, in particular some Bet v I proteins, are also increased during nodulation until levels similar as in the wild type are reached. Furthermore, the isoflavonoid levels are not increased, which might be associated to altered JA signaling or altered Bet v I protein accumulation. Lack of isoflavonoid induction might lead to elevated auxin shoot-to-root transport. Alternatively, there could be an increased shoot-to-root transport of isoflavonoids, since higher isoflavonoid levels have been detected in *nark* mutant roots (Cho and Harper, 1991). Solid black arrows indicate relationships between established components of the AON pathway reported in the literature. Red arrows and red crosses indicate changes observed in the *nark* mutant. Dashed black and red arrows or crosses indicate hypothetical relationships proposed based on the present data and previous literature, which have not been experimentally tested in this study. Created in BioRender.com.

### 4.2 Early nodulation in soybean roots involves coordinated signaling and metabolic reprogramming

Comparing the root proteome between 0 and 3 dpi resulted in a relatively moderate number (68) of differentially accumulating proteins, consistent with the early timing of the harvesting and the dynamic nature of nodulation signaling. In contrast, transcriptomics studies in *M. truncatula* and soybean, during the first stages of nodulation, reported a much greater number of deregulated genes, often using more localized tissue such as nodulated root regions or the zone of nodulation, instead of nodulated roots (Lohar et al., 2006; Hayashi et al., 2012). In addition to the differences in sampling material, this observed discrepancy is in line with the often limited correlation between transcriptomic data and protein abundance, especially during dynamic responses, due to post-transcriptional and/or post-translational regulation (Gunawardana and Niranjan, 2013).

The identification of proteins annotated as calcium signaling proteins, CCaMK and CDPK10, is in line with the activation of early symbiotic signaling following rhizobial perception (Santos et al., 2001; Ramu et al., 2002; Oldroyd and Downie, 2004). However, few identified proteins were linked to nodulation-specific pathways, suggesting that our results likely reflect early preparatory responses instead of nodulation-specific developmental processes. For example, differential abundances of proteins associated with cytoskeleton and membrane dynamics in the roots (SCAR2, LNP, 4CL9, ANXA13) could reflect active cellular reorganization during early nodulation. The identification of AMI1 (increased abundance) and ABCB9 (decreased abundance) proteins suggests a potential link between early nodulation and auxin signaling. Interestingly, the gene encoding ABCB9 was also reported to be downregulated at 3 dpi in an independent translatome study (Song et al., 2022). In line with this, proteins involved in tryptophan metabolism, a key precursor pathway for auxin biosynthesis, showed elevated abundance at 3 dpi. Together, these observations are consistent with a role for regulated auxin distribution and biosynthesis during early symbiosis rather than a global activation, consistent with previous findings in legumes (Pacios-Bras et al., 2003; Suzaki et al., 2012; Turner et al., 2013; Kohlen et al., 2018). Supporting these observations, the PPI network analysis grouped several differentially abundant proteins into different clusters associated with immune-related processes and tryptophan metabolism. Although the network included additional STRING-derived interactors that were not detected in our dataset, the resulting clusters suggest that the proteome changes observed can be part of coordinated biological processes rather than isolated responses.

Furthermore, several proteins that were identified as differentially abundant in soybean roots at 3 dpi were associated with nutrient signaling and metabolic regulation (PRS4, DSP4, DGAT2D), which may reflect a possible shift in energy production/allocation during the early stages of symbiosis to support the energy demands of the plant during such an interaction. Also, NRT2.4, a protein annotated as a high-affinity nitrate transporter and whose homologs have been implicated in nitrate uptake and redistribution within the plant (Kiba et al., 2012; Xu et al., 2024) showed an increased abundance at 3 dpi. Notably, our observation is consistent with an independent translatome dataset, showing differential regulation of NRT2.4 during the early steps of nodulation (Song et al., 2022). Increased NRT2.4 accumulation may reflect nitrate uptake during early symbiosis to support the plant’s demands in nitrogen. Nitrate also acts as a signaling molecule, hence NRT2.4 may contribute to fine-tuning nitrogen availability and signaling before the establishment of nitrogen fixation (Gautrat et al., 2021).

The present root proteomics dataset provides insights into the protein dynamics associated with early nodulation. Future inclusion of *nod4* root samples could further refine the distinction between general nodulation-associated responses and those specifically dependent on NARK-mediated regulation. An additional limitation of the root proteomics dataset was the exclusion of the 7 dpi samples due to the high proportion of missing protein abundance values. Imputation of these missing data had a substantial risk of false-positive protein identifications and differential abundance calls. As a result, our root analysis focused on early nodulation events. Future studies may allow the inclusion of later stages and provide a more comprehensive view of the transition from nodulation initiation to established systemic regulation.

### 4.3 Immunity and AON: what is the role of the Bet v I proteins?

Immunity needs to be strictly controlled to allow rhizobia infection and the development of nodules. Rhizobia have developed mechanisms to escape or alter plant defense responses (Berrabah et al., 2019). In our study, we observed changes in only two immunity-associated proteins (Bet15 and MOS14) in the roots. At the transcriptome level, transient induction of several host defense-related genes had been observed during early stages of rhizobial infection of soybean, Lotus, and Medicago (Kouchi et al., 2004; Lohar et al., 2006; Libault et al., 2010). Hence, defense response in the roots could already have been suppressed by the rhizobia at 3 dpi.

In contrast, in the leaves, several immunity-associated proteins were differentially regulated in the WT versus the *nark* mutant *nod4*. The basal abundance of several immunity-associated proteins appeared higher in Wm82 versus *nod4*, although protein abundances in the mutant increased following rhizobial inoculation and reached levels comparable to those observed in Wm82 (**Figure 7**). The differences in basal state between WT and *nod4* were observed not only for immunity-associated proteins but appear to represent a more general characteristic of the two genotypes. These baseline differences could be generally genotype-dependent, yet they might also be associated to AON signaling. Previous leaf transcriptome analyses in soybean during nodulation have indicated altered regulation of pathogenesis-related genes (Kinkema and Gresshoff, 2008; Reid et al., 2012). Two of these genes were overlapping with proteins in our data: a hypothetical BSP protein (C6SYB8) and a hypothetical enhanced disease susceptibility 1 (EDS1) protein (I1KCL7). Both genes were upregulated in the transcriptome 48 h after induction of AON by xylem sap feeding (Reid et al., 2012). However, in our data, the proteins were more abundant in *nod4* only, while in Wm82, protein abundance was stable (I1KCL7) or decreased (C6SYB8) over time.

Multiple Bet v I/Major latex domain-containing proteins were identified in our study. A characteristic of the Bet v I structure is a large hydrophobic cavity, where ligands such as phytohormones or flavonoids can bind. Although no specific biological function has been assigned to Bet v I proteins, this protein family has an important role in plant development and defense (Lakhssassi et al., 2020). Furthermore, overexpression of a cotton Bet v I protein (*Gh-MLP*) in Arabidopsis increased the total flavonoid content of leaves (Chen and Dai, 2010). The closest homologs of this protein in soybean were not detected (Glyma.15G274300) or did not show a significantly changed abundance (Glyma.09G102400). According to the phylogenetic tree of the Bet v I protein family in soybean, these homologs belonged to the same subclade as Bet1, Bet2, Bet15a, and Bet20, whose protein abundance changes were significant, though limited. For Bet15a, a significant increase between 0 and 3 dpi was observed in the WT roots as well.

In short, our data identified possible cross-talks between regulation of nodulation and immunity signaling, which is worthy of functional testing (**Figure 7**). Hypernodulation mutants with mutation in *nark*, or its homologs *har1* (*L. japonicus*) and *sunn* (*M. truncatula*), have been shown to be more susceptible to pathogen infection (Lohar and Bird, 2003; Tazawa et al., 2007; Ben et al., 2013). Correspondingly, we observed that mutation in *nark* affected the abundance of several immunity-associated proteins in a systemic manner. Our observations raise the possibility that NARK contributes to the maintenance of basal levels of immunity-associated proteins. Possibly, a NARK-independent pathway might be activated in *nark* mutants by rhizobia infection, leading to systemic changes in the abundance of immunity-associated proteins. Also, the role of the Bet v I proteins remains to be studied further. An interesting hypothesis would be the connection with isoflavonoid regulation, as suggested by Chen and Dai (2010), since we observed a deregulation of isoflavonoids in *nark* mutants.

### 4.4 Isoflavonoids and AON: how are they connected?

Similar as for the immunity-related proteins, also for isoflavonoid biosynthesis proteins a differential protein abundance profile was detected in *nod4* versus Wm82 leaves. In particular, the proteins identified were predicted to be involved in daidzein and genistein biosynthesis and conversion to derivatives. Metabolomic analysis of soybean leaves further implied a NARK*-*dependent regulation of isoflavonoid compounds. In general, flavonoids are well-known secondary metabolites, performing a multitude of functions, including cell growth regulation, attraction of pollinators and protection against biotic and abiotic stress (Rodriguez De Luna et al., 2020; Dias et al., 2021). A specific class of flavonoids includes the isoflavonoids, primarily known from legumes and functioning as pathogen-inhibiting phytoalexins and signaling molecules in the symbiotic interaction with rhizobia (Phillips and Kapulnik, 1995; Ferguson and Mathesius, 2003; Lozovaya et al., 2004; Eckardt, 2006; Subramanian et al., 2006; 2007; Shajib et al., 2012; Bentivenha et al., 2017). Daidzein and genistein are important isoflavonoids in soybean pathogen defense responses and are secreted in the rhizosphere to communicate with soil micro-organisms, including rhizobia (Sugiyama et al., 2017; Gómez et al., 2018; Sugiyama, 2019; Okutani et al., 2020; Zhang et al., 2022; Lario et al., 2024). The daidzein-derived formononetin and genistein-derived biochanin A are also involved in defense responses (Shajib et al., 2012; Murakami et al., 2014; Hu et al., 2021).

The isoflavone synthase (IFS) plays an important role in redirecting the flavonoid pathway to the isoflavonoid pathway and is described as the limiting step in determining isoflavonoid levels (Du et al., 2010; Sohn et al., 2021; Lyu et al., 2022a; Yang and Wang, 2024). In IFS RNAi soybean roots, virtually no isoflavonoids are detected (Subramanian et al., 2006). IFS was not identified in our data, however the second enzyme of the pathway, HID, showed a significantly increased abundance in *nod4*. HID appears to be another key limiting factor (Du et al., 2010). Interestingly, increased abundances of several isoflavonoid metabolites were observed in Wm82, despite relatively stable levels of the detected biosynthetic enzymes. Conversely, several proteins associated with isoflavonoid biosynthesis accumulated in *nod4* leaves without a corresponding increase in metabolite abundance. The contrasting patterns of enzyme abundance and metabolite accumulation imply a more complex regulation of isoflavonoid metabolism than can be explained by protein abundance alone, highlighting the need for further investigation. Although transcriptional regulation has been described, including by MYB transcription factors and miRNAs, the effect of post-translational regulation is not known (Gupta et al., 2017; Han et al., 2017; Anguraj Vadivel et al., 2019; Wang et al., 2024; Yang and Wang, 2024; Yang et al., 2026). Additionally, feedback inhibition is important as well, because genistein can inhibit IFS activity (Wang et al., 2026). Furthermore, altered abundance of metabolites might also be caused by a differential flow through the pathway. This could explain the different abundance profile that was observed for naringenin, the precursor of genistein. Naringenin might be consumed in Wm82 due to increased synthesis of genistein and biochanin A, while the metabolite accumulated in *nod4* where no increased genistein levels were observed. Possibly, the lack of increase in isoflavonoid abundance in *nod4* leaves might induce a compensatory response at the level of isoflavonoid biosynthesis enzymes, explaining their increased abundance.

Alternatively, the differential abundance of isoflavonoids in Wm82 and *nod4* mutant leaves might be a consequence of differential redistribution to other tissues, such as the roots (**Figure 7**). Flavonoid transport into vacuoles occurs via MATE transporters or vesicular trafficking (Petrussa et al., 2013; Zhao, 2015; Biala-Leonhard et al., 2021; Yang and Wang, 2024), however the mechanism of transporting flavonoids to different tissues, for example through the vasculature, has not been described. In inoculated roots of hypernodulation mutants, a significantly higher abundance of daidzein and genistein was observed compared to the WT Wm82 (Cho and Harper, 1991). This could be caused by altered biosynthesis in the root, since isoflavonoid metabolism genes are highly expressed in roots (Wang et al., 2024; Yang and Wang, 2024). However, grafting experiments have shown a NARK-dependent shoot control of root isoflavonoid levels in soybean (Abd-Alla, 2001). Hence, would the increased abundance of isoflavonoid biosynthesis proteins in *nod4* leaves lead to higher isoflavonoid levels, which are subsequently transported to the roots? Then, what is the role of the increased isoflavonoid content in Wm82 leaves? Maybe the metabolites play a role in auxin transport, since isoflavonoids are known auxin transport inhibitors (Peer et al., 2004; Peer and Murphy, 2007; Santelia et al., 2008; Ng et al., 2015). Interestingly, in *M. truncatula*, long distance auxin transport from shoot to root was higher in the *sunn* mutant. In the WT, this transport reduced after rhizobia inoculation, while no reduction was observed in the mutant (van Noorden et al., 2006). The observed increased isoflavonoid abundances in Wm82 leaves and not in *nod4*, corroborate these findings (**Figure 7**).

### 4.5 Jasmonic acid, isoflavonoids, and AON: are there parallels with induced systemic resistance?

Furthermore, there might be an intricate connection between AON signaling, JA, and isoflavonoids in soybean leaves. Methyl jasmonate (MeJA) treatment is known to induce isoflavonoid production (Jeong et al., 2018; Ye et al., 2025). Foliar application of MeJA was also shown to reduce nodule number in *L. japonicus*, both in the WT and the *har1* mutant (Nakagawa and Kawaguchi, 2006). Possibly, MeJA might act via the induction of isoflavonoids to inhibit nodulation. Transcriptional analyses of soybean leaves indicated a role of JA in AON signaling (Kinkema and Gresshoff, 2008; Reid et al., 2012). JA biosynthesis enzymes, such as lipoxygenases (LOX), were induced during AON signaling in the leaves and this induction was not detected in the *nark* mutant (Kinkema and Gresshoff, 2008; Reid et al., 2012). Two of the LOX enzymes overlapped with proteins in our study (I1KH70 and K7L7J7). They were significantly upregulated in Wm82 and *nod4* over time, hence the induction did not seem NARK-dependent. At 0 dpi, JA levels were found to be higher in *nark* mutant leaves compared to the WT, corresponding to earlier findings and suggesting that NARK might limit JA signaling in the absence of rhizobia (Seo et al., 2007; Kinkema and Gresshoff, 2008). Hence, the higher basal JA concentration in *nark* mutant leaves might be associated to the higher basal isoflavonoid levels we detected. Possibly, transient early changes in JA levels in WT leaves might be associated with the increased isoflavonoid levels at 7 dpi. However, the connection between JA dynamics, isoflavonoids and AON needs more experimental support. Future research should analyze additional (early) time points to evaluate the JA dynamics in soybean leaves during nodulation.

Moreover, AON was suggested to represent an induced systemic resistance-like mechanism, since microbial infections in local tissues activate priming of the plant via systemic signaling to defend against secondary infections (Zamioudis and Pieterse, 2012; Tonelli et al., 2020). JA is a known inducer of induced systemic resistance in plants (Dong, 1998; van Wees et al., 1999; Truman et al., 2007). Furthermore, JA induces isoflavonoids, which are tightly connected to immunity. Our data also suggest a potential association between Bet v I proteins and isoflavonoid metabolism (**Figure 7**). How are these elements interconnected and what is their association with AON and NARK signaling? Future research is required to unravel underlying molecular mechanisms in more detail.

## Data Availability

The mass spectrometry proteomics data have been deposited to the ProteomeXchange Consortium via the PRIDE partner repository with the dataset identifier PXD079829 (roots) and PXD078508 (leaves) (Perez-Riverol et al., 2025). The metabolomics data have been deposited to MetaboLights repository with the study identifier MTBLS14770 (Yurekten et al., 2024).

## Supplementary material

Supplementary Data Sheet 1 Gene expression data from RT-qPCR experiments.

Supplementary Data Sheet 2 Root proteomics data: significantly changing proteins.

Supplementary Data Sheet 3 Leaf proteomics data: significantly changing proteins and functional analysis.

Supplementary Data Sheet 4 Metabolomics data from the leaves: significantly changing metabolites and metadata with a special focus on (iso) flavonoids.

Supplementary Data Sheet 5 Leaf proteomics data: significantly changing proteins, including replicate 3 that was excluded from the initial analysis.
