# Supplementary material for: Systemic proteome remodeling in soybean during early nodulation: distinct responses in roots and leaves
Source: Front Plant Sci. 2026 Sep 16;17:1952260. doi: 10.3389/fpls.2026.1952260 (PMC13624086; doi:10.3389/fpls.2026.1952260)
Supplement: Supplementary file 1 [file SupplementaryFile1.docx]

**SUPPLEMENTARY DATA**

**Figure S1.** **Gene expression profile of AON markers in soybean root and leaf samples.** Gene expression was measured in four biological replicates (R1 to R4, 3 to 5 plants each) and displayed using separate bars. Gene expression data are represented as fold gene expression, calculated based on the 2‑^ΔΔCt^ method (Livak and Schmittgen, 2001), normalized to the reference genes *GmATPs* and *GmCons6* and subsequently to the non-inoculated control (0 dpi). **(A-B)** *RIC1* and *RIC2* expression in roots. **(C-D)** *PremiR2111c* and *premiR2111f* expression in leaves. **(E-F)** *TML1a* and *TML1b* expression in roots. Statistical significance was evaluated with Welch Anova and Games-Howell post-hoc analysis, *: P value < 0.05, **: P value < 0.01 (Games and Howell, 1976). Arrows indicate the overall trend that was observed for every gene.

**Figure S2.** **Gene expression profile of AON markers in soybean root and leaf samples that were used for proteomics.** Gene expression data (n = 1) are represented as fold gene expression, calculated based on the 2‑^ΔΔCt^ method (Livak and Schmittgen, 2001), normalized to the reference genes *GmATPs* and *GmCons6* and subsequently to the non-inoculated wild type sample (Wm82, 0 dpi). **(A)** *RIC1* and *RIC2* expression in roots. **(B)** *PremiR2111c* and *premiR2111f* expression in leaves. **(C)** *TML1a* and *TML1b* expression in roots.

**Figure S3****. Principal component analysis (PCA) plot of the shotgun proteomics leaf samples.** Samples cluster per repeat, rather than per genotype or timepoint. R1, repeat 1; R2, repeat 2; R3, repeat 3.

**Figure S4.** **PPI networks of deregulated proteins in roots during early nodulation of wild-type soybean. (A)** A protein–protein interaction network was constructed using the STRING database (Szklarczyk *et al.*, 2023). Cytoscape was used for visualization and analysis to identify densely connected clusters, potentially representing co-functional protein complexes, or pathways. Functional enrichment analysis was performed for each identified cluster using the STRING plugin. Each cluster was annotated as Cluster 1, 2, and 3 and within are listed the proteins names identified. Lines indicate interactions between proteins and the color corresponds to the LogFC of each protein identified in the shotgun dataset. Orange labels indicate of the proteins with increased abundance (LogFC>0.5), blue labels indicate of the proteins with decreased abundance (LogFC<0.5). **(B)** PPI network identified via STRING DB. The proteins in the table include proteins the shotgun analysis dataset and predicted interactors via STRING DB. The LogFC column is indicating the changes observed in our shotgun analysis dataset where applicable.

**Figure S5.** **Protein profile plots of immunity-related proteins and isoflavonoid biosynthesis proteins in Wm82 and *nod4* leaves during nodulation (replicate 2). (A)** Protein profile plot of immunity-related proteins. **(B)** Protein profile plot of Bet v I domain-containing proteins. Red, green and black lines represent Bet v I proteins from separate subclades, the colors correspond to the phylogenetic tree in Figure 5C. **(C)** Protein profile plot of (iso)flavonoid biosynthesis proteins. Protein names can be found in Dataset S3. The log_2_ LFQ values (Intensity) of replicate 2 are represented, the profile plots with values from replicate 1 can be found in Figures 5 and 6.

**Figure S6.** **PCA plot from metabolomics analysis in soybean leaves and boxplots depicting relative abundances of selected compounds. (A)** PCA plot. Pooled samples contain material of all samples and represent an internal control. **(B-E)** Boxplots representing the normalized intensities of selected compounds: **(B)** formononetin (m/z: 269.08, RT: 9.43 min). **(C)** glyceollin (m/z: 361.11, RT: 11.80 min). **(D)** biochanin A (m/z: 285.08, RT: 11.95 min). **(E)** jasmonic acid (m/z: 211.13, RT: 11.114 min).

**Figure S7.** **Abundance profiles of different genistein isomers in soybean leaves during nodulation.** Boxplots representing the normalized intensities of genistein isomers with m/z: 271.06. **(A)** Retention time (RT): 11.21 min. **(B)** RT: 5.93 min. **(C)** RT: 8.71 min. **(D)** RT: 7.50 min. **(E)** 7.26 min.

**Table S1.** **Composition of Soli, the nutrient solution used for growing soybean.**

| **Component** | **Concentration** |
| --- | --- |
| KNO_3_ | 0.505 g/l |
| KH_2_PO_4_ | 0.68 g/l |
| CaCl_2_ x 2 H_2_O | 1.825 g/l |
| MgSO_4_ x 7 H_2_O | 0.615 g/l |
| K_2_SO_4_ | 1.0875 g/l |
| EDTA_2_Na_2_Fe (Sigma E6760) | 0.205 g/l |
| H_3_BO_3_ | 0.275 g/l |
| MnSO_4_ x H_2_O | 0.155 g/l |
| KCl | 0.25 g/l |
| ZnSO_4_x7H_2_O | 0.025 g/l |
| (NH_4_) 6 Mo_7_O_24_ x 4 H_2_O | 0.025 g/l |
| CuSO_4_ x 5 H_2_O | 0.0125 g/l |
| H_2_SO_4_ | 0.0125 ml/l |

**Table S2.** **Primers used in this study for RT-qPCR.**

| **Name** | **Sequence** | **Reference** |
| --- | --- | --- |
| *GmATPs-fw* | GCGATTCTTAAGCCAGCCTTT | (Reid *et al.*, 2011) |
| *GmATPs-rev* | ACACACCCTGGAAACTGGTGA | (Reid *et al.*, 2011) |
| *GmCons6-fw* | AGATAGGGAAATGGTGCAGGT | (Libault *et al.*, 2008) |
| *GmCons6-rev* | CTAATGGCAATTGCAGCTCTC | (Libault *et al.*, 2008) |
| *GmRIC1-fw* | CAAATGCAACAATGGCTACTCG | This study |
| *GmRIC1-rev* | GCCATGGAGATTACTAGCCTGC | This study |
| *GmRIC2-fw* | TCGGGTATTCACCTATCAAAGCG | This study |
| *GmRIC2-rev* | CGTGGTGGAGCTCTCACATTA | This study |
| *GmmiR2111c-fw* | GGTGGAGTTAATATTATTGAGTGAGGACA | (Zhang *et al.*, 2021) |
| *GmmiR2111c-rev* | CACATCCAAATTCTATTGATTGAACCG | (Zhang *et al.*, 2021) |
| *Gmmir2111f-fw* | GTAGCTGCTATCATACTAGACAGTTGA | (Zhang *et al.*, 2021) |
| *Gmmir2111f-rev* | CTTATATGAATTTCTCTACATGGATGGTC | (Zhang *et al.*, 2021) |
| *GmTML1a-fw* | CATGTTTGTGTGTTGATTATTCTC | This study |
| *GmTML1a-rev* | ATTTCTACCCCAAGGCTAC | This study |
| *GmTML1b-fw* | TCATGGCATGTTTGTGTGTTGA | This study |
| *GmTML1b-rev* | GCCAATGCTCCCCCAATATG | This study |

**Table S3.** **PPI network identified via STRING DB.** The proteins in the table include proteins from the shotgun analysis dataset and predicted interactors via STRING DB. The LogFC column indicates the changes observed in our shotgun analysis dataset where applicable.

| **Network proteins and interactors** | | | |
| --- | --- | --- | --- |
| **Protein.IDs** | **Protein.Description** | **Gene.Names** | **LogFC** |
| A0A0R0FNH1 | CBS domain-containing protein | Glyma.17G129600 | -4,48 |
| A0A0R0G2R3 | NADPH OXIDASE | Glyma.15G182000 |  |
| A0A0R0GIC4 | Fe2OG dioxygenase domain-containing protein | Glyma.13G048500 | 0,71 |
| A0A0R0I3B3 | Pre-mRNA polyadenylation factor Fip1 domain-containing protein | Glyma.09G035800 |  |
| I1J550 | Phosphoribosyltransferase domain-containing protein | Glyma.01G026800 | 2,36 |
| I1JUS7 | Eukaryotic translation initiation factor 3 subunit L | Glyma.04G080800 | -4,66 |
| I1KCN9 | Calcium-dependent protein kinase (CDPK10) | Glyma.06G189600 | 2,79 |
| I1KD31 | Cleavage stimulation factor 50 kDa subunit | Glyma.06G205000 |  |
| I1L0D3 | 30-kDa cleavage and polyadenylation specificity factor 30 | Glyma.09G022200 | -2,37 |
| I1M4U5 | RRM domain-containing protein | Glyma.13G334200 | -1,56 |
| I1MT65 | FAD-binding FR-type domain-containing protein | Glyma.17G078300 |  |
| I1N388 | Elongation factor P | Glyma.18G213900 | 2,94 |
| I1N583 | W2 domain-containing protein | Glyma.18G292300 | -3,77 |
| I1NHQ7 | Tyrosine specific protein phosphatases | Glyma.20G190400 | 0,73 |
| I1NHY2 | RRM domain-containing protein | Glyma.20G198900 | 0,82 |
| I1NIY1 | Amidase domain-containing protein | Glyma.20G232900 | 2,03 |
| K7KME9 | FAD-binding FR-type domain-containing protein | Glyma.05G021100 |  |
| K7L0Z1 | glutamate--tRNA ligase | Glyma.07G124000 | -2,04 |
| K7L881 | calcium/calmodulin-dependent protein kinase (DMI3 protein) | Glyma.08G227200 | 2,68 |
| K7LCU5 | Pre-mRNA polyadenylation factor Fip1 domain-containing protein | Glyma.09G097600 |  |
| K7M1M4 | Tyrosinase copper-binding domain-containing protein | Glyma.13G242400 | 1,1 |
| K7M5M9 | FMR1-interacting protein 1 conserved domain-containing protein | Glyma.14G084400 |  |
| K7MBA3 | Pre-mRNA polyadenylation factor Fip1 domain-containing protein | Glyma.15G140800 |  |
| K7MD15 | Pre-mRNA polyadenylation factor Fip1 domain-containing protein | Glyma.15G205600 |  |
| K7MNN7 | FMR1-interacting protein 1 conserved domain-containing protein | Glyma.17G240500 |  |
| K7MPW9 | FACT complex subunit | Glyma.18G044600 | 3,81 |

**Table S4.** **PPI network enrichment analysis of cluster 1 (Node 1) of the identified network.** The PPI network was identified by the STRING database and the Cytoscape software was used for visualization and further analysis. The MCODE plugin was applied to identify densely connected clusters, potentially representing co-functional protein complexes or pathways, and a functional enrichment analysis was performed for each identified cluster using the STRING plugin.

| **Cluster 1 with interactors** | | | | | | |
| --- | --- | --- | --- | --- | --- | --- |
| **# background genes** | **# genes** | **Category** | **Description** | **FDR value** | **P-value** | **Genes** |
| 68 | 7 | STRING Clusters | Processing of Intronless Pre-mRNAs, and Ribonuclease J, domain 2 | 4.48E-16 | 4.71E-20 | A0A0R0I3B3\|I1KD31_SOYBN\|I1L0D3_SOYBN\|I1NHY2_SOYBN\|K7LCU5_SOYBN\|K7MBA3_SOYBN\|K7MD15_SOYBN |
| 36 | 6 | STRING Clusters | mRNA cleavage factor complex, and Fip1 motif | 8.26E-15 | 3.48E-18 | A0A0R0I3B3\|I1KD31_SOYBN\|I1L0D3_SOYBN\|K7LCU5_SOYBN\|K7MBA3_SOYBN\|K7MD15_SOYBN |
| 167 | 7 | Reactome Pathways | RNA Polymerase II Transcription Termination | 1.76E-14 | 2.01E-17 | A0A0R0I3B3\|I1KD31_SOYBN\|I1M4U5_SOYBN\|I1NHY2_SOYBN\|K7LCU5_SOYBN\|K7MBA3_SOYBN\|K7MD15_SOYBN |
| 50 | 6 | COMPARTMENTS | mRNA cleavage factor complex | 3.47E-14 | 2.15E-17 | A0A0R0I3B3\|I1KD31_SOYBN\|I1L0D3_SOYBN\|K7LCU5_SOYBN\|K7MBA3_SOYBN\|K7MD15_SOYBN |
| 29 | 5 | STRING Clusters | mRNA cleavage factor complex, and Fip1 motif | 3.63E-12 | 3.43E-15 | A0A0R0I3B3\|I1KD31_SOYBN\|I1L0D3_SOYBN\|K7MBA3_SOYBN\|K7MD15_SOYBN |
| 903 | 8 | COMPARTMENTS | Nucleoplasm | 3.92E-12 | 4.85E-15 | A0A0R0I3B3\|I1KD31_SOYBN\|I1L0D3_SOYBN\|I1M4U5_SOYBN\|I1NHY2_SOYBN\|K7LCU5_SOYBN\|K7MBA3_SOYBN\|K7MD15_SOYBN |
| 32 | 5 | COMPARTMENTS | mRNA cleavage and polyadenylation specificity factor complex | 3.92E-12 | 5.38E-15 | A0A0R0I3B3\|I1L0D3_SOYBN\|K7LCU5_SOYBN\|K7MBA3_SOYBN\|K7MD15_SOYBN |
| 4 | 4 | Pfam | Fip1 motif | 3.68E-11 | 1.21E-14 | A0A0R0I3B3\|K7LCU5_SOYBN\|K7MBA3_SOYBN\|K7MD15_SOYBN |
| 60 | 5 | Reactome Pathways | Processing of Intronless Pre-mRNAs | 4.47E-11 | 1.02E-13 | A0A0R0I3B3\|I1KD31_SOYBN\|K7LCU5_SOYBN\|K7MBA3_SOYBN\|K7MD15_SOYBN |
| 68 | 5 | Reactome Pathways | Processing of Capped Intronless Pre-mRNA | 5.41E-11 | 1.85E-13 | A0A0R0I3B3\|I1KD31_SOYBN\|K7LCU5_SOYBN\|K7MBA3_SOYBN\|K7MD15_SOYBN |
| 4 | 4 | InterPro Domains | Pre-mRNA polyadenylation factor Fip1 domain | 1.07E-10 | 1.21E-14 | A0A0R0I3B3\|K7LCU5_SOYBN\|K7MBA3_SOYBN\|K7MD15_SOYBN |
| 4 | 4 | InterPro Domains | FIPS3/FIPS5-like | 1.07E-10 | 1.21E-14 | A0A0R0I3B3\|K7LCU5_SOYBN\|K7MBA3_SOYBN\|K7MD15_SOYBN |
| 763 | 7 | Reactome Pathways | Processing of Capped Intron-Containing Pre-mRNA | 1.6E-10 | 7.27E-13 | A0A0R0I3B3\|I1KD31_SOYBN\|I1M4U5_SOYBN\|I1NHY2_SOYBN\|K7LCU5_SOYBN\|K7MBA3_SOYBN\|K7MD15_SOYBN |
| 24 | 4 | STRING Clusters | mRNA cleavage factor complex, and Fip1 motif | 3.35E-9 | 3.53E-12 | A0A0R0I3B3\|I1KD31_SOYBN\|I1L0D3_SOYBN\|K7MBA3_SOYBN |
| 907 | 7 | GO Biological Process | mRNA processing | 1.17E-8 | 2.42E-12 | A0A0R0I3B3\|I1KD31_SOYBN\|I1L0D3_SOYBN\|I1NHY2_SOYBN\|K7LCU5_SOYBN\|K7MBA3_SOYBN\|K7MD15_SOYBN |
| 1899 | 7 | Reactome Pathways | RNA Polymerase II Transcription | 7.26E-8 | 4.13E-10 | A0A0R0I3B3\|I1KD31_SOYBN\|I1M4U5_SOYBN\|I1NHY2_SOYBN\|K7LCU5_SOYBN\|K7MBA3_SOYBN\|K7MD15_SOYBN |
| 2349 | 7 | Reactome Pathways | Metabolism of RNA | 2.65E-7 | 1.81E-9 | A0A0R0I3B3\|I1KD31_SOYBN\|I1M4U5_SOYBN\|I1NHY2_SOYBN\|K7LCU5_SOYBN\|K7MBA3_SOYBN\|K7MD15_SOYBN |
| 2439 | 7 | Reactome Pathways | Gene expression (Transcription) | 2.95E-7 | 2.35E-9 | A0A0R0I3B3\|I1KD31_SOYBN\|I1M4U5_SOYBN\|I1NHY2_SOYBN\|K7LCU5_SOYBN\|K7MBA3_SOYBN\|K7MD15_SOYBN |
| 3240 | 8 | GO Molecular Function | RNA binding | 3.37E-7 | 1.3E-10 | A0A0R0I3B3\|I1KD31_SOYBN\|I1L0D3_SOYBN\|I1M4U5_SOYBN\|I1NHY2_SOYBN\|K7LCU5_SOYBN\|K7MBA3_SOYBN\|K7MD15_SOYBN |
| 3903 | 8 | GO Biological Process | Gene expression | 8.94E-7 | 5.73E-10 | A0A0R0I3B3\|I1KD31_SOYBN\|I1L0D3_SOYBN\|I1M4U5_SOYBN\|I1NHY2_SOYBN\|K7LCU5_SOYBN\|K7MBA3_SOYBN\|K7MD15_SOYBN |
| 200 | 4 | KEGG Pathways | mRNA surveillance pathway | 1.59E-6 | 1.19E-8 | I1KD31_SOYBN\|I1L0D3_SOYBN\|I1M4U5_SOYBN\|I1NHY2_SOYBN |
| 12516 | 8 | GO Cellular Component | Nucleus | 0.0058 | 6.37E-6 | A0A0R0I3B3\|I1KD31_SOYBN\|I1L0D3_SOYBN\|I1M4U5_SOYBN\|I1NHY2_SOYBN\|K7LCU5_SOYBN\|K7MBA3_SOYBN\|K7MD15_SOYBN |

**Table S5.** **PPI network enrichment analysis of cluster 2 (Node 2) of the identified network.** The PPI network was identified by the STRING database and the Cytoscape software was used for visualization and further analysis. The MCODE plugin was applied to identify densely connected clusters, potentially representing co-functional protein complexes or pathways, and a functional enrichment analysis was performed for each identified cluster using the STRING plugin.

| **Cluster 2 with interactors** | | | | | | |
| --- | --- | --- | --- | --- | --- | --- |
| **# background genes** | **# genes** | **Category** | **Description** | **FDR value** | **P-value** | **Genes** |
| 354 | 5 | KEGG Pathways | Plant-pathogen interaction | 1.42E-9 | 1.07E-11 | A0A0R0G2R3\|I1KCN9_SOYBN\|I1MT65_SOYBN\|K7KME9_SOYBN\|K7L881_SOYBN |
| 24 | 4 | STRING Clusters | NADPH oxidase Respiratory burst, and Calcium-dependent protein kinase activity | 2.4E-9 | 2.52E-13 | A0A0R0G2R3\|I1KCN9_SOYBN\|I1MT65_SOYBN\|K7KME9_SOYBN |
| 367 | 5 | InterPro Domains | EF-hand domain pair | 1.13E-7 | 1.28E-11 | A0A0R0G2R3\|I1KCN9_SOYBN\|I1MT65_SOYBN\|K7KME9_SOYBN\|K7L881_SOYBN |
| 529 | 5 | GO Molecular Function | Calcium ion binding | 2.04E-7 | 7.84E-11 | A0A0R0G2R3\|I1KCN9_SOYBN\|I1MT65_SOYBN\|K7KME9_SOYBN\|K7L881_SOYBN |
| 19 | 3 | GO Molecular Function | NAD(P)H oxidase H2O2-forming activity | 6.9E-7 | 5.3E-10 | A0A0R0G2R3\|I1MT65_SOYBN\|K7KME9_SOYBN |
| 28 | 3 | GO Molecular Function | Ferric-chelate reductase activity | 1.01E-6 | 1.55E-9 | A0A0R0G2R3\|I1MT65_SOYBN\|K7KME9_SOYBN |
| 18 | 3 | Pfam | Respiratory burst NADPH oxidase | 1.39E-6 | 4.58E-10 | A0A0R0G2R3\|I1MT65_SOYBN\|K7KME9_SOYBN |
| 26 | 3 | Pfam | Ferric reductase like transmembrane component | 1.53E-6 | 1.26E-9 | A0A0R0G2R3\|I1MT65_SOYBN\|K7KME9_SOYBN |
| 26 | 3 | Pfam | FAD-binding domain | 1.53E-6 | 1.26E-9 | A0A0R0G2R3\|I1MT65_SOYBN\|K7KME9_SOYBN |
| 24 | 3 | Pfam | Ferric reductase NAD binding domain | 1.53E-6 | 1.01E-9 | A0A0R0G2R3\|I1MT65_SOYBN\|K7KME9_SOYBN |
| 17 | 3 | InterPro Domains | NADPH oxidase Respiratory burst | 1.74E-6 | 3.92E-10 | A0A0R0G2R3\|I1MT65_SOYBN\|K7KME9_SOYBN |
| 26 | 3 | InterPro Domains | FAD-binding 8 | 3.34E-6 | 1.26E-9 | A0A0R0G2R3\|I1MT65_SOYBN\|K7KME9_SOYBN |
| 25 | 3 | InterPro Domains | Ferric reductase, NAD binding domain | 3.34E-6 | 1.13E-9 | A0A0R0G2R3\|I1MT65_SOYBN\|K7KME9_SOYBN |
| 26 | 3 | InterPro Domains | Ferric reductase transmembrane component-like domain | 3.34E-6 | 1.26E-9 | A0A0R0G2R3\|I1MT65_SOYBN\|K7KME9_SOYBN |
| 51 | 3 | InterPro Domains | Riboflavin synthase-like beta-barrel | 1.26E-5 | 8.53E-9 | A0A0R0G2R3\|I1MT65_SOYBN\|K7KME9_SOYBN |
| 55 | 3 | InterPro Domains | FAD-binding domain, ferredoxin reductase-type | 1.35E-5 | 1.06E-8 | A0A0R0G2R3\|I1MT65_SOYBN\|K7KME9_SOYBN |
| 59 | 3 | InterPro Domains | Ferredoxin-NADP reductase (FNR), nucleotide-binding domain | 1.44E-5 | 1.3E-8 | A0A0R0G2R3\|I1MT65_SOYBN\|K7KME9_SOYBN |
| 274 | 3 | KEGG Pathways | MAPK signaling pathway - plant | 7.96E-5 | 1.2E-6 | A0A0R0G2R3\|I1MT65_SOYBN\|K7KME9_SOYBN |
| 279 | 3 | GO Molecular Function | Peroxidase activity | 4.7E-4 | 1.26E-6 | A0A0R0G2R3\|I1MT65_SOYBN\|K7KME9_SOYBN |
| 18 | 2 | InterPro Domains | Cytochrome b245, heavy chain | 0.0012 | 1.22E-6 | A0A0R0G2R3\|K7KME9_SOYBN |
| 306 | 3 | InterPro Domains | EF-Hand 1, calcium-binding site | 0.0015 | 1.66E-6 | A0A0R0G2R3\|I1KCN9_SOYBN\|K7L881_SOYBN |
| 326 | 3 | InterPro Domains | EF-hand domain | 0.0016 | 2.01E-6 | A0A0R0G2R3\|I1KCN9_SOYBN\|K7L881_SOYBN |
| 601 | 3 | UniProt Keywords | Calcium | 0.0043 | 1.24E-5 | A0A0R0G2R3\|I1KCN9_SOYBN\|K7L881_SOYBN |
| 85 | 2 | GO Molecular Function | Calmodulin-dependent protein kinase activity | 0.0057 | 2.39E-5 | I1KCN9_SOYBN\|K7L881_SOYBN |
| 81 | 2 | GO Molecular Function | Calcium-dependent protein serine/threonine kinase activity | 0.0057 | 2.17E-5 | I1KCN9_SOYBN\|K7L881_SOYBN |
| 5952 | 5 | GO Biological Process | Cellular response to stimulus | 0.0134 | 1.38E-5 | A0A0R0G2R3\|I1KCN9_SOYBN\|I1MT65_SOYBN\|K7KME9_SOYBN\|K7L881_SOYBN |
| 1380 | 3 | UniProt Keywords | Oxidoreductase | 0.0255 | 1.5E-4 | A0A0R0G2R3\|I1MT65_SOYBN\|K7KME9_SOYBN |
| 2608 | 4 | COMPARTMENTS | Plasma membrane | 0.0371 | 2.3E-5 | A0A0R0G2R3\|I1MT65_SOYBN\|K7KME9_SOYBN\|K7L881_SOYBN |

**Table S6.** **PPI network enrichment analysis of cluster 3 (Node 3) of the identified network.** The PPI network was identified by the STRING database and the Cytoscape software was used for visualization and further analysis. The MCODE plugin was applied to identify densely connected clusters, potentially representing co-functional protein complexes or pathways, and a functional enrichment analysis was performed for each identified cluster using the STRING plugin.

| **Cluster 3 with interactors** | | | | | | |
| --- | --- | --- | --- | --- | --- | --- |
| **# background genes** | **# genes** | **Category** | **Description** | **FDR value** | **P-value** | **Genes** |
| 7 | 2 | STRING Clusters | Amidase activity, and Flavin-binding monooxygenase-like | 0.0022 | 2.31E-7 | A0A0R0GIC4\|I1NIY1_SOYBN |
| 83 | 2 | KEGG Pathways | Tryptophan metabolism | 0.003 | 2.28E-5 | A0A0R0GIC4\|I1NIY1_SOYBN |
